# Bridging Themes: Short Protein Segments Found in Different Architectures

**DOI:** 10.1093/molbev/msab017

**Published:** 2021-01-27

**Authors:** Rachel Kolodny, Sergey Nepomnyachiy, Dan S Tawfik, Nir Ben-Tal

**Affiliations:** 1 Department of Computer Science, University of Haifa, Haifa, Israel; 2 Department of Biomolecular Sciences, Weizmann Institute of Science, Rehovot, Israel; 3 George S. Wise Faculty of Life Sciences, Department of Biochemistry and Molecular Biology, Tel Aviv University, Tel Aviv, Israel

**Keywords:** protein space, protein evolutionary patterns, ancestral segments, bridging themes

## Abstract

The vast majority of theoretically possible polypeptide chains do not fold, let alone confer function. Hence, protein evolution from preexisting building blocks has clear potential advantages over ab initio emergence from random sequences. In support of this view, sequence similarities between different proteins is generally indicative of common ancestry, and we collectively refer to such homologous sequences as “themes.” At the domain level, sequence homology is routinely detected. However, short themes which are segments, or fragments of intact domains, are particularly interesting because they may provide hints about the emergence of domains, as opposed to divergence of preexisting domains, or their mixing-and-matching to form multi-domain proteins. Here we identified 525 representative short themes, comprising 20–80 residues that are unexpectedly shared between domains considered to have emerged independently. Among these “bridging themes” are ones shared between the most ancient domains, for example, Rossmann, P-loop NTPase, TIM-barrel, flavodoxin, and ferredoxin-like. We elaborate on several particularly interesting cases, where the bridging themes mediate ligand binding. Ligand binding may have contributed to the stability and the plasticity of these building blocks, and to their ability to invade preexisting domains or serve as starting points for completely new domains.

## Introduction

Over the course of 3.7 billion years of protein evolution protein segments of varying lengths mutated, duplicated, and recombined (Eck and Dayhoff 1966; Grishin 2001a; Aravind et al. 2002; Nepomnyachiy et al. 2017; Alva and Lupas 2018). Contemporary proteins may hold hints to these “historical” events. A likely scenario is that they evolved by duplication and fusion of short polypeptides with at least marginal stability, and weak biological functionality, sufficient for their preference over random alternatives. By mining protein databases (Hubbard et al. 1997; Berman 2000; Greene et al. 2007; Cheng et al. 2014), one can computationally search for traces of the evolutionary events that shaped the current protein universe, such as mutations, duplications, and recombinations of short protein segments (Dokholyan et al. 2002; Chothia 2003; Alva et al. 2010; Nepomnyachiy et al. 2010, 2017; Edwards and Deane 2015; Franklin et al. 2018). Convergence is also a scenario that may result in sequence similarity. But because sampling a specific sequence (even as short as a few dozen residues) from the vast number of possible sequences is an extremely low probability event, when sequence segments of sufficient similarity are detected, common ancestry (homology) is the more likely scenario (Lupas et al. 2001). In practical terms, there are accurate, fast, and sensitive sequence aligners (e.g., HHSearch; Soding 2005 or HMMER; Finn et al. 2011) to identify similarities indicative of common descent. Most of the observed homology among current-day proteins may reflect relatively recent events. Nonetheless, there is also hope of finding protein segments that are relics of primordial, ancestral peptides that gave rise to what is now two or more separate lineages. These segments would typically comprise conserved and functionally critical parts of contemporary proteins (Laurino et al. 2016; Longo et al. 2020). In the words of Eck and Dayhoff in their seminal 1966 paper, this is due to “natural selection which severely inhibits any change to an (ancient) well-adapted system on which several other essential components depend” (Eck and Dayhoff 1966).

The most widely recognized form of shared segments are protein units of ∼100 residues called domains (Chothia 2003; Vogel et al. 2004; Cheng et al. 2014; Scaiewicz and Levitt 2018; Yu et al. 2019). The definition of what is a domain varies (Kelley and Sternberg 2015), emphasizing structure (Wetlaufer 1973), or sequence (Finn et al. 2014). In the structure-based definition, domains are segments that fold independently to standalone (and even globular) entities (Kessel and Ben-Tal 2018). Using this definition, protein space includes overall different domains with similar substructures (Harrison et al. 2002; Fernandez-Fuentes et al. 2010), which might reflect biophysical constraints on the protein chain (Finkelstein and Ptitsyn 1987; Orengo et al. 2001; Skolnick et al. 2014). In the alternative definition, emphasizing sequence, domains are commonly found protein segments that share significant sequence similarity with each other (Finn et al. 2014). Indeed, using this definition reveals domains as evolutionary entities found in different combinations or, equivalently, protein space has many instances of overall different protein chains with shared domains (Chothia 2003; Forslund et al. 2019). The domain databases use the latter definition and group domains of the same evolutionary lineage (Murzin et al. 1995; Orengo et al. 1997; Marchler-Bauer et al. 2011; Cheng et al. 2014; Finn et al. 2014). When studying protein evolution, the initial focus was on standalone domains, because unlike short segments that cannot even fold, these can readily serve as evolutionary building blocks. This description, however, makes one wonder about the emergence of domains, and whether segments that are smaller than a domain, yet cannot fold or function on their own, played a role in that.

Indeed, sequence similarity among segments shorter than domains has also been described (Lupas et al. 2001; Söding and Lupas 2003; Friedberg and Godzik 2005; Kolodny et al. 2006; Goncearenco and Berezovsky 2011; Alva et al. 2010; Nepomnyachiy et al. 2014, 2017). In fact, we have observed that the number of statistically significant similar segments increases with the decrease in their length (number of amino acids; Nepomnyachiy et al. 2017). Presumably, the proteins in the ancient protein universe were shorter, and the long period of time that passed offered their sequences many “copy-paste” opportunities. Consequently, short segments that show meaningful sequence homology are candidates for such ancient segments. Similarity between short segments can be classified into two types. The first includes series of repeated or amplified (similar) copies of a given segment in the same protein chain (Alva and Lupas 2018), suggesting emergence by duplication and fusion. Indeed, repeated segments can be identified from the internal symmetry in the sequence of the protein chain. The example that Eck and Dayhoff identified early on, was a short repeating segment in ferredoxin binding an iron–sulfur cluster (Eck and Dayhoff 1966; see Romero et al. 2016 for a retrospective view of this discovery). More recent examples include the ancient double β-hairpins and longer elements identified in the outer membrane beta barrels (Remmert et al. 2010; Franklin et al. 2018; Nanda 2019), and the repeating β-blade forming β-propellers (Chaudhuri et al. 2008; Smock et al., 2016). The second type comprises homologous segments found in different contexts, namely in proteins that are deemed to have no common evolutionary origin. Prominent examples are the KH motif (Grishin 2001b; Lupas et al. 2001), the short segments with the same function in different SCOP folds (Goncearenco and Berezovsky 2015) and the Fuzzle database (Ferruz et al. 2020). Most relevant to this study is Alva, Söding, and Lupas’s curated set of 40 ancient segments that are shared among domains of different SCOP folds (Alva et al. 2015).

In a previous study, we systematically documented similar protein segments that are shared between different proteins, referring to them as “themes” and to the individual occurrences within each theme as “variations” (Nepomnyachiy et al. 2017). Here, we take advantage of these themes to describe a set of yet unknown “bridging themes,” namely homologous protein segments that are found in different sequential and structural contexts. The challenge in constructing such a set is that most similarity is detected among homologous domains, rendering the shared origin of such themes trivial (as these domains share common ancestry). To avoid these, we look only for cases where the variations of a given theme are found among domains whose overall sequences and structures are different, thus excluding shared ancestry of the entire domain. That a theme is shared between two current-day domains that are thought to have evolved independently suggests that this theme may have played a significant role in the emergence of these domains. Specifically, a shared theme may reflect common ancestry, although its precise role may vary. Assuming that modern proteins evolved from short polypeptides, a single ancestral fragment could extend by accretion or fusion to different other segments, and ultimately give rise to two different domains each of a different fold. Alternatively, a segment can be coopted from a preexisting domain, and fused to another segment, or duplicated to generate a new fold. At this stage, these two scenarios cannot be distinguished. We thus dub these themes, bridging themes, because regardless of the precise scenario, which they are currently found within the seed of 2-fold(s)/domain(s) that are deemed independent evolutionary lineages attests to their ability to fit in different environments.

To detect these bridging themes, we search for sequence similarity in a (non redundant) set of domains that are classified as evolutionarily distinct (Cheng et al. 2014). In addition, we verify that beyond the shared theme, the rest of the domain sequences are not homologous. We find 525 such bridging themes, spanning 73 different folds, including the most ancient, pre-LUCA (Last Universal Common Ancestor) enzyme folds Rossmanns, P-loops, TIM-barrels, and flavodoxins (Wang et al. 2006, 2011). The identified themes uncover many previously unknown potential evolutionary relationships, including ones that relate these ancient folds to each other. In approximately half the cases, the context change is also accompanied by significant alteration in the structure of the theme itself.

## Results

### Detecting Bridging Themes

We identify themes shared between nonhomologous protein domains—cases where similar protein segments are found in two different contexts. More specifically, the segments should be similar to each other because they are detected using an adequately low HHSearch (Soding 2005) E-value, and their similarity is verified by a high-sequence alignment score. Yet their contexts are different as judged by structure- and sequence-based criteria. The structure context is deduced from the ECOD classification of the domains. The five levels of the ECOD hierarchy, the so-called A.X.H.T.F groups, classify domains based on their structure and shared evolutionary origin (Cheng et al. 2014; Schaeffer et al. 2016). The top A (architecture) level does not indicate evolutionary relationships but rather is based on the secondary structure content. The remaining levels of the hierarchy, from the X (possible homology) level downward group domains based on presumed common ancestry. Domains with the same X classification (denoted X-groups) designate a distinct fold, and are possibly of common origin, yet more evidence is needed to establish whether they did in fact descend from a common ancestor. The next levels—H (homology), T (topology), and F (family)—group domains with increasing levels of overall sequence and structural similarity, thus clearly indicating common ancestry (Schaeffer et al. 2016). To be conservative, we focus on themes shared between different X-groups, that is, between domains that emerged independently even by the most lax ECOD definition of independent evolutionary lineages. The sequence context was also examined by applying, in addition to the above ECOD classification–based filter, another filter based on sequence similarity. We look for cases where the best possible alignments of the domain segments flanking the shared theme (before the theme or/and after it) are poor, as these are indicative of a different sequence context.

**Fig. 1.**
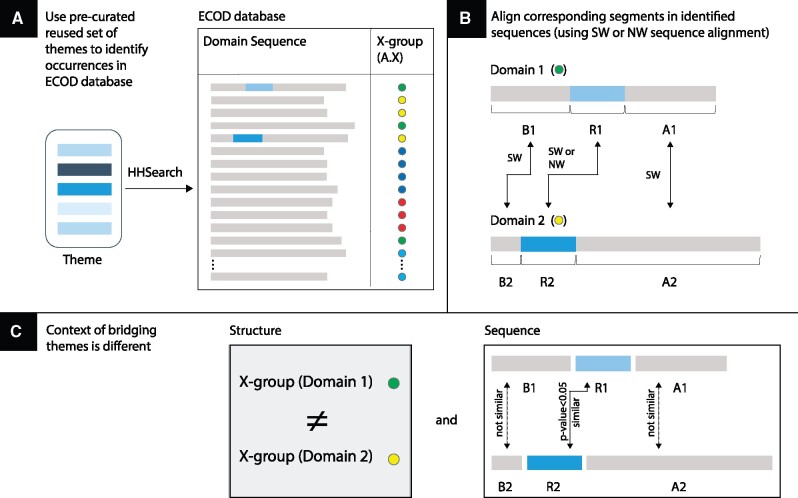
Overview of the process of identifying bridging themes. (*A*) We rely on a precurated set of 12,769 themes (Nepomnyachiy et al. 2017) (one theme is represented here by shades of blue) and use HHSearch to search for segments in the ECOD database that exhibit sequence homology. Each ECOD is characterized by its X-group (a unique number), represented here by a colored dot. (*B*) Themes that appear in two domains each belonging to a different X-group (domains 1 and 2) are further analyzed by calculating the optimal sequence alignments of three segments: An alignment between the matched recurring parts (the themes, R1 vs. R2) using the local Smith–Waterman or the global Needleman–Wunsch algorithms, and local Smith–Waterman alignments between the segments before (B1 vs. B2) and the segments after (A1 vs. A2) the theme. (*C*) Overall, we search for events in which the context of the shared theme differs: The structure context differs if the domains are from different X-groups, and the sequence context differs if the local alignments (B1 and B2) and (A1 and A2) have low similarity.

Figure 1 illustrates the search process that we use to detect bridging themes. Using HHSearch, we search for shared themes in an ECOD database of domains that has been reduced in redundancy to 70% sequence identity (see Materials and Methods for details). Figure 1*A* shows this search for one instance, highlighting two domains in the database (domains 1 and 2) with segments that match the query with low *E* values indicative of homology (<10^−3^). We then select only those pairs of domains that belong to different ECOD X-groups. These pairs are broken to three parts (fig. 1*B*): the recurring part, the part before it, and the part after. We align the matching recurring parts to each other using a local (Smith–Waterman [SW]) or a global (Needleman–Wunch [NW]) aligner. Figure 1*C* summarizes the properties that we verify prior to including a pair in the set of curated bridging themes, which comprises 525 themes, spanning 73 different ECOD X-groups, or folds. The structure context is different, and the sequence context differs because the SW alignment scores of the parts before and after is low, and yet the shared theme is of common origin.

### General Properties of the Bridging Themes Set

**Fig. 2.**
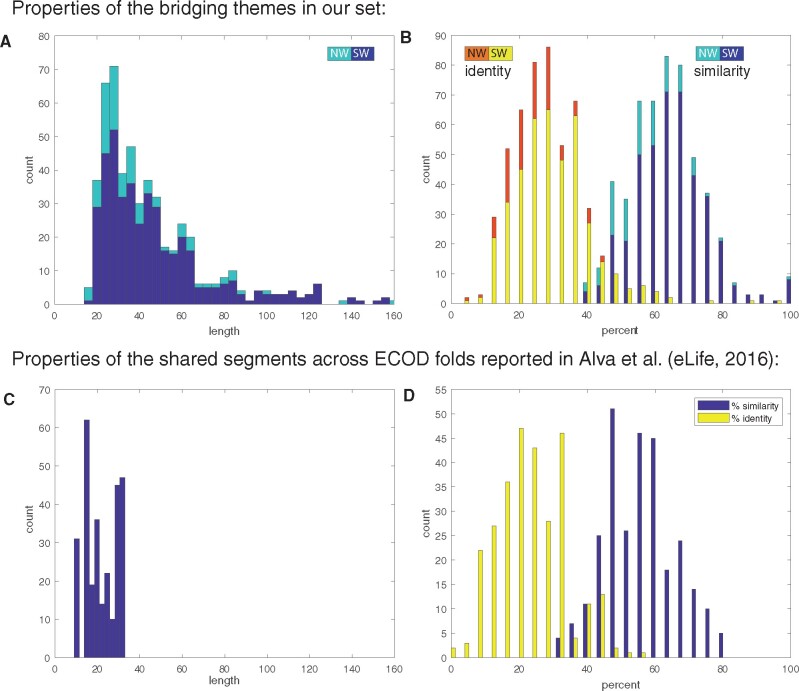
The cumulative properties of the bridging themes in our data set versus the fragment set of Alva et al. (2015). The distributions of length (*A*) and sequence identity/similarity (*B*) in our data set. By design, our themes are longer than 20, and their mean length was found to be ∼49 residues. The mean sequence similarity is ∼64%, and the mean sequence identity is ∼30%. The distributions of length (*C*) and sequence identity/similarity (*D*) for the pairs of ancient fragments in the set of Alva et al. Shown is the subset of all pairs that span two ECOD X-groups (*n* = 286). The mean length of the recurring segment in this set is ∼22 residues, the mean sequence similarity is ∼56% and the mean sequence identify is ∼24%. In general, the two data sets have similar criteria for selecting segments of shared origin, although our data set is somewhat more conservative, and accordingly our themes are longer and with higher sequence similarity/identity.

Figure 2*A* and *B* shows the distributions of the length, and of sequence identity, or similarity, of the bridging themes in our set. For completeness, supplementary figure 1S, Supplementary Material online, relates these measures and *E* values, to one another. As dictated by our search procedure, the themes are longer than 20 residues (save ten cases, where the alignment results in fewer residues), and because we search for similarities between domains of average length of 100 amino acids, the themes are generally shorter than 80 residues, with a mean length of ∼49. The high mean sequence similarity of ∼64% and mean sequence identity of ∼30%, as well as the low *P* values of the alignment scores, all indicate that these themes likely reflect common ancestry. In contrast, the segments before and after the bridging theme fail to show significant homology. Specifically, we align the corresponding parts before and after the recurring segments using a local SW aligner. In 85% (444) of the pairs, either the alignment is too short (<20 residues), or the best alignment results in sequence identity lower than 25%. For the segments after, in 88% (464) of the pairs, either the alignment is too short (<20 residues), or sequence identity is lower than 25%. In the remaining cases (which are left in the set but not described), the local SW sequence alignment in the part before or after is not significantly worse than that of the shared theme part. However, because this is a local alignment, it is not contiguous with the recurring part. It is possible, however, that the (evolutionary linked) recurring segments are longer (and detectable by allowing additional longer gaps).

The chains covering our themes include 121,749 residues in total, out of which 31,085 comprise the bridging themes. Supplementary figure 2S*A*, Supplementary Material online, compares the percent of different classes of secondary structure, and supplementary figure 2S*B*, Supplementary Material online, compares the percentages (i.e., a normalized histogram) of the solvent accessibility values. In both cases, the distributions are similar, that is, the themes are not unique in their secondary structure nor their solvent accessibility.

### Structural Similarity of the Variations of the Bridging Themes

**Fig. 3.**
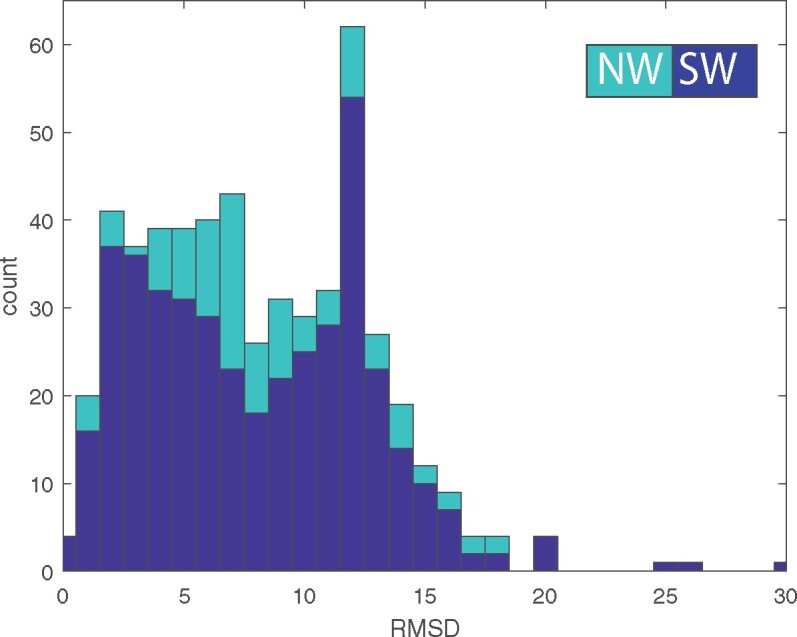
The histogram of RMSD values between the variations of the bridging themes in our data set. The values for the Smith–Waterman (local) alignments are shown in blue, and for the Needlman–Wunch (global) alignments are in light blue. The global average is ∼8 Å RMSD, yet the histogram appears to be a mixture of two distributions, one of structurally similar themes (characterized by lower RMSD values), and one of structurally dissimilar themes in spite of their high-sequence similarity. Using a 6 Å RMSD threshold, 39% of these sequence variations are structurally similar, and 61% have different conformations. When fitting a mixture of two Gaussian distributions, we find that 28% of the pairs of variations that share the same theme also share a similar conformation (averaging ∼3.3 Å) and 72% do not (averaging ∼9.8 Å).

We did not restrict our search to cases where the variations of the bridging themes are structurally similar (i.e., within the two domains where the themes are found). Figure 3 shows the distribution of their Root Mean Square Deviation (RMSD) (over the matching C-alpha atoms) and these indeed vary. The average RMSD over the whole data set is 8 Å. Using a 6 Å RMSD threshold, the structures of the recurring themes in 39% (203) of the pairs are similar, and 61% (322) are not. Because RMSD is sensitive to outliers, and hence may be overestimating the variations, we used additional measures to quantify the structural similarity: We structurally aligned the matching domain segments with TM align (Zhang and Skolnick 2005): Supplementary figure 3S*A* and *B*, Supplementary Material online, shows the histograms of TM scores and RMSDs of the aligned residues. TM scores are on a 0–1 scale, where scores >0.5 indicate of the same fold and <0.3 corresponds to random structural similarity. In our bridging themes set, 30% have a score <0.3, and only 25% have a score >0.5. Supplementary figure 3S*C*–*F*, Supplementary Material online, shows the histogram of percent agreement of secondary structure assignment (by the algorithm Define Secondary Structure of Protein, DSSP), dRMSD (distance RMSD), and percent contact map change of the aligned residues (see Materials and Methods for details). These measures also indicate that our bridging themes set includes pairs with different structures. The structural variation is consistent with the sequence homology being the outcome of common ancestry, rather than convergence due to structural constrains. It appears that one of the qualities of the bridging themes is that their structures can vary in accordance with their different contexts.

### Enrichment of Binding Residues within the Bridging Themes

For approximately half of the domains in our data set, we can identify a binding function from their PDB structures. We identify binding residues (and the domains that include them) in one of two ways: 1) residues within 4.5 Å of a ligand and 2) residues listed in the BioLip database (Yang et al. 2012) (see Materials and Methods for details). Because the bridging themes cover only some of the domain’s residues, binding residues may be either within the theme or not. Had the binding residues been chosen uniformly and at random from all domain residues, we would expect that (on average) the proportion of binding residues in a bridging theme out of all binding residues is the same as the relative proportion of bridging theme residues in the domain. Supplementary figure 4S, Supplementary Material online, shows that when comparing these ratios, there are many cases above the diagonal line, that is, the binding residues are more likely to be within a bridging theme than what is expected from the lengths of these themes. The total number of residues in the binding domains are 30,822 (BioLiP data set) and 38,724 (4.5 Å data set); the total number of residues in the themes in these domains are 11,533 (BioLiP) and 14,135 (4.5 Å). The ratio between the two is 0.37 for both sets. The total number of binding residues within these domains are 2,400 (BioLiP) and 3,843 (4.5 Å); the total number of binding residues that are in a bridging theme is 1,321 (BioLiP) and 2,062 (4.5 Å). The ratio between the two is 0.54–0.55, which is larger than 0.37, suggesting that bridging themes are more likely to relate to function compared with their flanking segments. However, it does not preclude other structural roles of bridging themes, especially because ligand binding involves many amino acids beyond those that directly mediate the interaction with the ligand.

### Network Views of the Bridging Themes

**Fig. 4.**
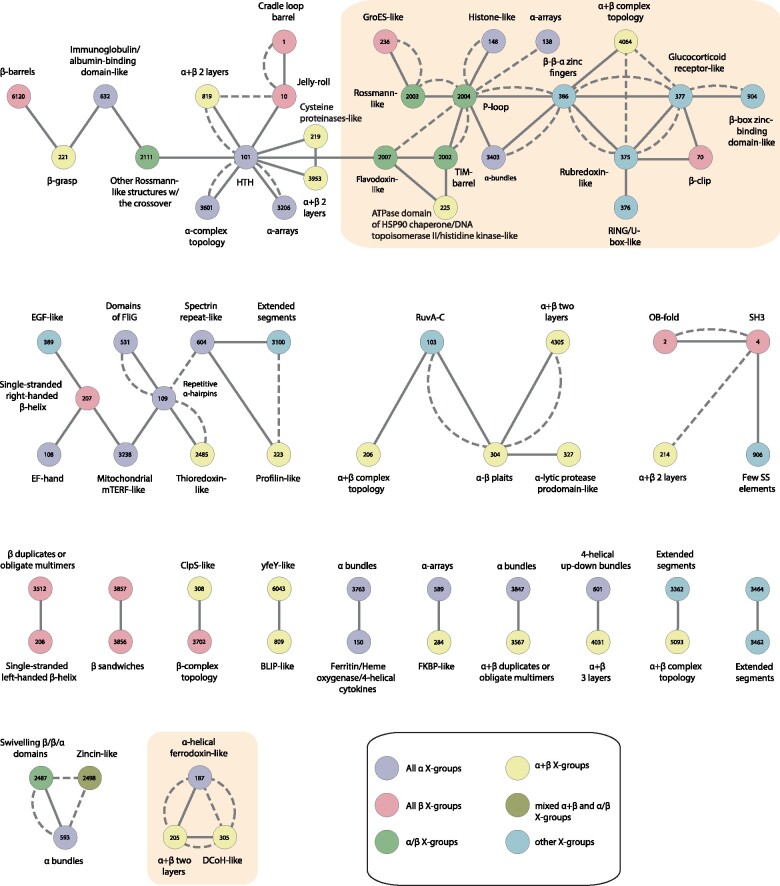
The overview network representing the 73 ECOD X-groups with bridging themes that are shared between them. The ECOD X-group number is listed in the node, and the corresponding name is listed next to it. The colors correspond to the X-group classes (see legend). Edges connect pairs of X-groups that share a common theme. The shared themes were found either by aligning the relevant segments in the two domains using a local (SW) or a global (NW) alignment. If the optimal local (SW) alignment of the shared theme that is longer than 20 residues, the edge is a solid line, otherwise, it is a dashed line. Connected components that are further discussed are highlighted with yellow background. The connected component on the upper row is further described in figure 5 and supplementary figure 1S, Supplementary Material online, and the connected component in the bottom row includes the examples in figures 6 and 7.

**Fig. 5.**
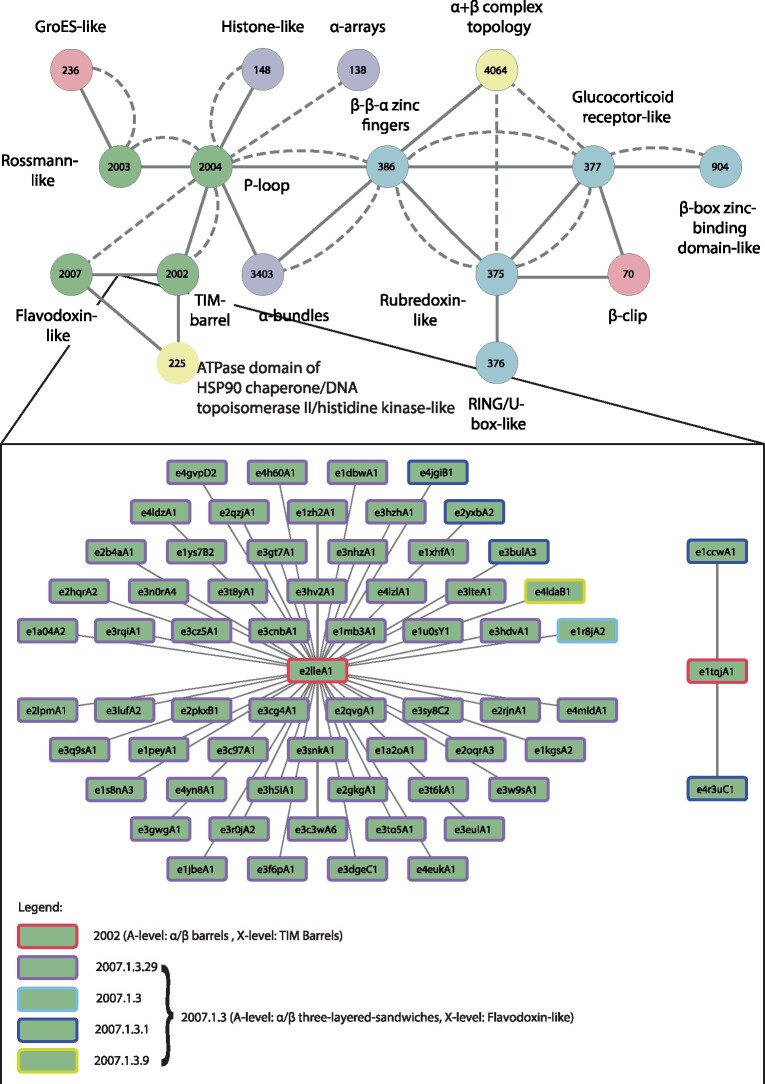
An example of the nested network describing the domains and their shared themes relating ECOD X-groups TIM-Barrels (2002) and flavodoxin-like (2007). The nested network (lower panel) expands on an edge between these two X-groups in the overview network (a snippet of this network is shown in the upper panel). Within the nested network, each domain is described by a node, and edges connect pairs of domains, one from each X-group. The nodes’ background is colored by their A level classification using the same color coding as in figure 4 (in this case, green, as they are all α/β domains). The nodes’ boundaries are colored to differentiate their lower-level classifications, with arbitrarily chosen colors. Most of the shared themes are between the same TIM-barrel domain e2lleA1 and various flavodoxin-like domains. However, there are also shared themes between another TIM-barrel domain, e1tqjA1, and two flavodoxin-like domains.

We detected 525 instances of bridging themes among 73 different X-groups belonging to 17 (of the possible 20) different architectures (A-groups). Supplementary table 1S, Supplementary Material online, lists these X-groups, and the number of domains in each. The number of members in the X-groups varies between 1 and 69 (ECOD ID 2007, flavodoxin-like), with an average of 7. The X-groups are identified by their ECOD ID: for example, 101 for HTH or 2004 for the P-loops. This set includes all-alpha, all-beta, alpha/beta, alpha + beta, and mixed alpha/beta and alpha + beta, suggesting that bridging themes are shared throughout the entire protein universe. We organize the bridging themes as networks in two levels. Figure 4 shows the first, overview level. Nodes represent ECOD X-groups, colored according to their ECOD A-group classification, and edges connect the two different X-groups among which the detected theme is shared. We find themes shared between X-groups from almost all class combinations, and in particular relating alpha/beta and alpha + beta proteins to either all-alpha or all-beta ones. More strikingly, some themes are shared between all-alpha and all-beta proteins. As X-groups contain many domains, a pair of connected X-groups, that is, an edge in the overview network, may represent more than one instance; an instance being a shared theme relating one of the possible pairs of non redundant domains (one from the first X-group, and the other from the second; see Materials and Methods for details). Thus, for each edge in the overview network, we organize all its instances as a separate (nested) network. Nodes in the nested network (fig. 5) represent the domains from the two X-groups, and edges in the nested network connect domains with variations of the same theme. A nested network may include alternative representatives from the 70% Non Redundant (NR) data set from different X-groups, which are similar to each other within each X-group (fig. 5). We use Cytoscape (Saito et al. 2012)/CytoStruct (Nepomnyachiy et al. 2015) to visualize the networks; the Cytoscape session is in the supplementary material, and online (https://trachel-srv.cs.haifa.ac.il/rachel/bridgingthemes/overview.html).

The most extensively connected component in the overview network (fig. 4, uppermost) relates five alpha/beta X-groups (Rossmann-like, Rossmann-like w/crossover, flavodoxin-like, a P-loop domains-like, and a TIM barrel), seven all-alpha X-groups (alpha bundles, two alpha arrays, Histone-like, alpha complex topology, immunoglobulins, and HTH), five all-beta X-group (GroES-like, jelly-rolls, cradle-loop barrels, and beta-clips), six groups of alpha + beta complex topology (alpha + beta two layers, beta-grasp, Cysteine proteinases-like, and ATPase domain of HSP90 chaperone/DNA topoisomerase II/histidine kinase-like), and five X-groups of few secondary structure elements. Interestingly, many of these, and the alpha/beta X-groups in particular, are considered to have been present in the LUCA (Caetano-Anollés et al. 2007; Ma et al. 2008; Edwards et al. 2013; Alva et al. 2015), suggesting that we traced events relating domains that evolved particularly early and that are not detectable by global domain sequence similarity. Supplementary figure 5S, Supplementary Material online, zooms in on part of this connected component to show examples of a shared theme for some of the pairs of connected X-groups. The most gregarious X-groups in this subset in terms of shared themes are the P-loops (ID 2004) and the HTH (ID 101). That they share themes with many other X-groups is consistent with previous estimates that they are ancient (Edwards et al. 2013).

Figure 5 shows an example of the network of the themes shared between the flavodoxin-like (2007) and the TIM-barrel (2002) X-groups; this is the network nested in the edge connecting the nodes 2007 and 2002. The domains in these two X-groups cluster into two connected components. The first is star-like, with the TIM-barrel domain e2lleA1 at its center, connected to no fewer than 60 different flavodoxin-like domains. The single domain protein e2lleA1 (PDB ID 2lle) was engineered by Höcker and coworkers as a copy-paste instance between these two X-groups (Eisenbeis et al. 2012). That we detect this artificially designed protein validates our approach. In fact, because we allow sequence variability in the theme, we identify different flavodoxin domains, similar (at least in part) to the one used in the design of 2lle. The domains belong to the ECOD T-group 2007.1.3 (flavodoxin-like/Class I glutamine amidotransferase-like/CheY-like): 55 of them in the F-group 2007.1.3.29 (Response_reg), 3 in 2007.1.3.1 (B12-binding), 1 in 2007.1.3.9 (OKR_DC_1_N_like); e4ldaB1 is unmapped. Interestingly, the second connected component in figure 4 reveals themes shared between the naturally occurring flavodoxin domains e1ccwA1 and e4r3uC1 (both, F-group 2007.1.3.1 B12-binding), and the TIM-barrel domain e1tqjA1 (see supplementary fig. 6S, Supplementary Material online, for more details), suggesting an evolutionary event independent of the artificial design.

### Relation to Alva et al.’s Ancient Fragments

Alva et al. (2015) recently curated a set of 40 ancient protein fragments. Their set covers all previously documented cases as well as instances that were not known before. We downloaded their set and assigned the ECOD classification to their fragments. Their set includes 40 fragments, where each fragment is described as an Multiple Sequence Alignment (MSA) of sequence segments from different proteins. Twenty-three of these fragments span different ECOD X-groups. The number of sequences in the MSAs of each of their fragments varies from 2 (e.g., fragment #30 in their set) to 20 (fragment #1 in their set). For the purpose of comparing to our set of bridging themes, we consider a subset of sequence segment pairs that are in the same fragment, that is, are aligned to each other in the MSA of that fragment, yet the ECOD X-groups of the domains of these segments differ; there are 286 such pairs in their data set. For every such pair, we measured the number of aligned residues, and percent similarity and identify, after aligning the reported segments with the global NW algorithm. Figure 2*B* and *D* shows the distribution of these values. Compared with our set of bridging themes, their ancient fragments are shorter, between 9 and 33 residues (mean of ∼22) versus 16–380 (mean of ∼49) in ours. The average sequence identity (24%) and similarity (56%) in their set is also lower than in our set (30% and 64%, respectively).

Supplementary figure 7S, Supplementary Material online, shows an overview network derived from the abovementioned 23 fragments in Alva et al.’s set that are classified to different ECOD X-groups (44 X-groups in total). Only 17 X-groups, and 3 pairs of X-groups, are found in both sets—ours and Alva et al.’s—(supplementary figs. 7S and 8S, Supplementary Material online). That the overlap between the results is relatively small and is due to the different search strategies used. Our strategy makes explicit use of the expectation that the segments are themes, that is, commonly used parts, as elaborated in the “Comparison to Alva et al.’s paper” section in Methodology.

### Examples of Newly Identified Bridging Themes

**Fig. 6.**
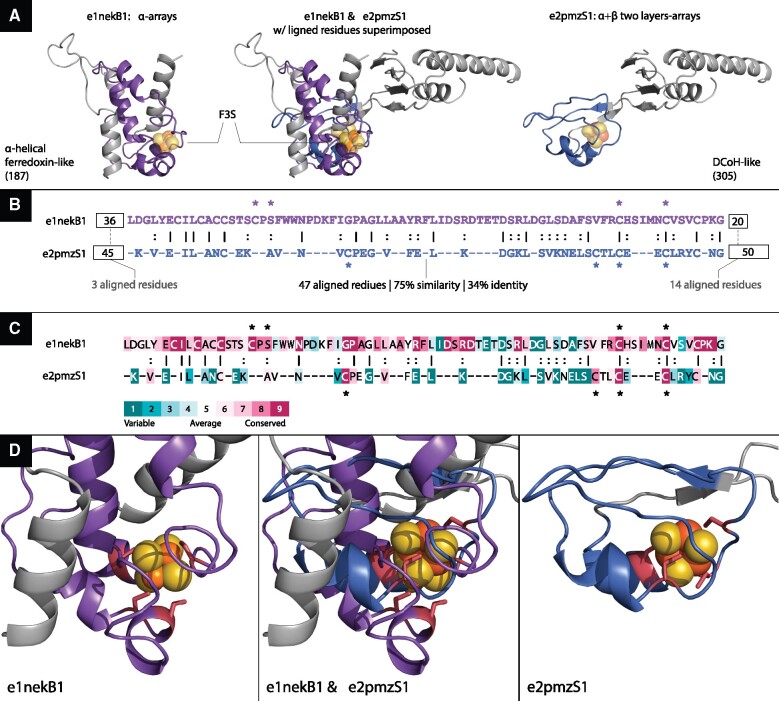
An F3S-binding theme shared between domain e1nekB1 from X-group 187 and domain e2pmzS1 from X-group 305. (*A*) The overall structures of these two domains is different: e1nekBa (left) is an alpha-helical ferredoxin-like from the all-alpha class, and e2pmzS1 (right) is a DCoH-like from the α + β class. In the middle, are the two structures in which this theme is found, in the best possible superimposition of the 47 aligned theme residues, and their two corresponding F3S clusters shown as orange (iron) and yellow (sulfur) spheres. We see that the iron–sulfur clusters reside in equivalent locations. (*B*) When aligning the shared theme (magenta) there are 47 equivalent residues, with overall sequence similarity of 75% and sequence identity of 34%. The residues binding the iron–sulfur (F3S) clusters are marked by asterisks: in e1nekB1, there are three cysteine residues (C159, C206, and C212) plus a serine (S161), and in e2pmzS1, there are four cysteine residues (C183, C203, C206, and C209). The sequence context of the two variations of this theme are different: The best local alignment of the ∼40 residues before the theme has only three aligned residues, and the sequences after the theme share 14 aligned residues (of 20 residues in e1nekB1 and 50 in e2pmzS1). (*C*) ConSurf evolutionary analysis of the corresponding proteins (1nek and 2pmz) and their 150 respective homologs. As can be seen, the binding residues are highly conserved (three with maximal conservation score 9, and one with conservation score 8 in both proteins). (*D*) Zooming-in on the domains’ iron–sulfur clusters. The two structures superimposed in the middle, and each one separately on both sides. The cluster-binding residues are shown in red and their side chains are shown as sticks.

For concreteness, we briefly describe only some of the bridging themes. A notable evolutionary link depicts a theme shared by ECOD e1nekB1 and e2pmzS1 of the alpha-helical ferredoxin-like (187) and DCoH-like (305) X-groups, respectively (fig. 6). The structures are positioned so that the shared segments are superimposed based on the sequence alignment, although as can be seen they vary in structure (for clarity, the individual structures are shown on the sides; fig. 6*A*). The sequences of these two variations of the same theme are homologous (47 aligned residues with 34% sequence identity and 75% similarity; fig. 6*B*), yet their sequence context, namely the sequence segments before and after the shared theme, are not (fig. 6*C*). Also, not only are the overall structures of the domains different, but also even the structures of the theme itself are quite different (optimal C-alpha RMSD is 9.7 Å). Although detected in two evolutionary distinct ECOD X-groups, both variations bind an F3S iron–sulfur cluster, further corroborating their shared evolutionary origin. Moreover, upon optimal structural superimposition of the two variations, their ligands reside in the spatial vicinity of one another (figs. 6*A* and *D*). Interestingly, even though the two variations share the same F3S ligand, their binding modes are somewhat different: F3S binding is coordinated by four cysteine residues in e2pmzS1 versus three cysteine residues and a serine in e1nekB1 (fig. 6*C*). Despite these differences, and the different structure of the shared theme in the two domains, two of the cysteine residues are well aligned. Accordingly, ConSurf (Ashkenazy et al. 2016) evolutionary analysis shows that the four residues that mediate F3S binding in both e1nekB1 and in e2pmzS2 are highly conserved among the homologs of the two respective proteins (1nek and 2pmz; fig. 6*C*). It is noteworthy that the two variations of the theme differ from each other in their conservation pattern. The e1nekB1 variation is much more evolutionarily conserved (among 1nek’s homologues) than that of e2pmzS2, reflecting the effect of years of evolution in different context: succinate dehydrogenase, where the iron–sulfur cluster is essential for enzymatic function versus RNA polymerase, where it mostly plays a supporting role in stability and folding (Baranovskiy et al. 2018).

**Fig. 7.**
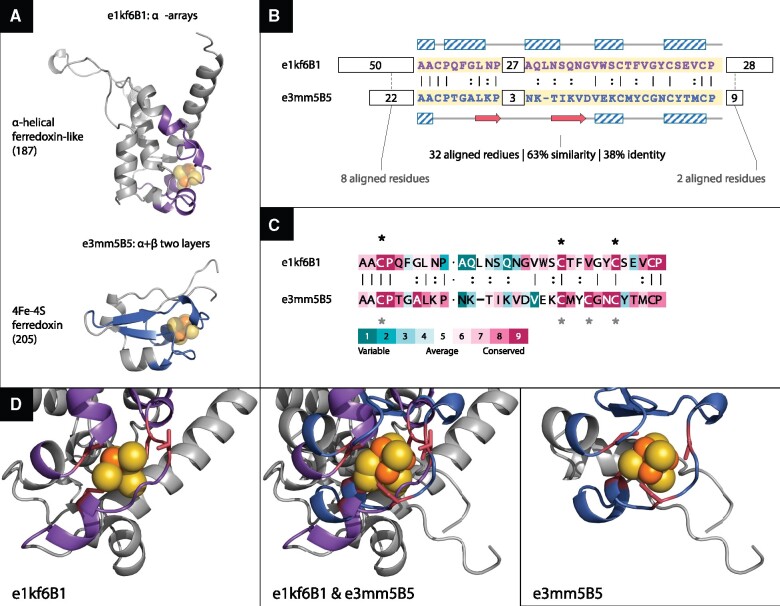
An iron–sulfur cluster-binding theme shared between e1kf6B1 (a ferredoxin-like domain of the all-alpha class) and e3mm5B5 (a 4Fe–4S ferredoxin domain of the α + β class). Fifteen representative instances connecting five domains in the 187 group (e1e7pB1, e1kf6B1, e1nekB1, e3cf4A4, and e3vr8B1), and 13 domains in the 205 group (e1fxdA2, e1hfeL2, e2fgoA1, e2gmhA1, e2v2kA1, e2wscC1, e2xsjB8, e2xsjD9, e2zvsA1, e3eunA1, e3j16B5, e3mm5B5, and e4id8A1) are found between these two X-groups. (*A*) The structures of the two domains, including the recurring theme (magenta), are different. Two bound ligands (FS3 within e1kf6B1 and SF4 within e3mm5B5) are shown in atoms-spheres representation (iron in orange and sulfur in yellow). (*B*) Although alignment of the shared theme suggest common ancestry (38% identity and 63% similarity over 32 residues), the secondary structures of the two variations differ: the α-helices are marked by diagonally patterned blocks and the β-strands by red arrows. Accordingly, the parts before and after the shared theme have different lengths and secondary structures and cannot be successfully aligned. (*C*) ConSurf analysis of the corresponding proteins (1kf6 and 3mm5) and (150 of) their respective homologs show that the binding residues (marked with asterisks) are highly conserved. In e1kf6B1, C158, C204, and C210 were assigned a conservation grade of 9 and T205 (the residues in proximity to the cluster but may not be ligating it directly) a grade of 7. In e3mm5B5, all four cysteine residues (C220, C241, C244, and C247) were assigned a grade of 9. (*D*) Zooming-in on the binding sites. The iron–sulfur cluster is shown as orange–yellow spheres, the themes are colored in magenta, the cluster-binding residues are in red, and the side chains of the binding residues are shown in sticks. In the middle, the two structures are shown after superimposing the aligned theme residues, and on the left/right, the structures are shown individually. Superimposing the aligned variations of the theme in the two domains (middle), results also in their F3S and SF4 ligands being well aligned.

Figure 7 shows an example of a theme shared between domains from ECOD X-groups 187 an alpha-helical ferredoxin-like (α-arrays ECOD A-group), and 205 a 4fe-4s ferredoxin (α + β-two layers ECOD A-group). Between these two X-groups, we found 15 representative instances, connecting 5 domains in the 187 group and 13 domains in the 205 group. The figure shows one of these instances, where a theme of 32 amino acids appears in the α-helical e1kf6B1 and in the α + β domain e3mm5B5. Despite the high-sequence similarity between the two variations of this theme, their structures are very different. Specifically, the secondary structure of the residues in the two contexts differ—there are only helices in the former and helixes surrounding a beta hairpin in the latter (figs. 7*B* and *D*). These differences are in accordance with the same theme being embedded in two very different sequence contexts (fig. 7*B*). Interestingly, both domains also bind a ligand—F3S in e1kf6B1 and SF4 in e3mm5B5—encapsulated by some of the residues of the theme and positioned similarly with respect to it. Figure 7*D* zooms in on the region in space where the variations encapsulate the ligands, showing how well the ligands align to each other upon superimposition of their respective variations. The sequence of two variations of the shared theme align well with 63% sequence similarity and 38% identity (fig. 7*B*). However, the segment inserted in the middle of the theme differs between the two variations (fig. 7*B*): in e1kf6B1, there are 27 residues and in e3mm5B5, only 3. Their difference indicates that both insertions may have taken place after the emergence of the theme. In e1kf6B1, the inserted 27 residues form a helical-hairpin, with many of its residues (180–192) exposed. Oddly enough, this insertion is in the folding core of the domain, suggesting that is has been key to the formation of the domain. Accordingly, homologues of e1kf6B1 (in the F-group 187.1.1.5) share this inserted segment, suggesting that it was one of the defining events in the diversion of this group of domains. The flanking segments do not align well at all: Before the matched theme, there are 50 residues in e1kf6B1 and 22 in e3mm5B5, and yet the optimal local alignment matches only 8 residues. After the matched theme, there are 28 and 9 residues, respectively, but the optimal local alignment matches only 2 residues. ConSurf’s evolutionary analysis of the two corresponding proteins (1kf6 and 3mm5) within the context of their respective homologues shows that the residues that mediate iron–-sulfur cluster binding, and several other residues, are highly conserved (fig. 7*C*). Of these, the three cysteine residues in e1kf6B1 align with their equivalents in e3mm5B5, reflecting that despite different ligands (F3S vs. SF4), these two variations resemble each other in their binding modes.

Shorter versions of iron–sulfur cluster-binding motifs were reported based on sequence and structure similarity searches (Lupas et al. 2001; Krishna et al. 2006; Alva et al. 2015). In particular, the bridging theme of figure 7 is closely related to fragment #18 in Alva et al.’s set, which connects X-groups 187 and 205. However, fragment #18 is about half the length. Two of the 14-residue segments that it includes are from the domain e2bs2B1, a homologue of e1kf6B1. The first segment overlaps with the part before the gap in our bridging theme (its last 5 residues align to the first 5 residues of our bridging theme), and the second segment overlaps with the last 13 residues of our bridging theme. One might argue that the high (more than 30%) sequence identities in the shared themes described in figures 6 and 7 have emerged by convergence (Krishna et al. 2006). Indeed, the need to coordinate the cluster in a way that will enable proper electron transfer function imposes not only cysteines as the ligating (coordinating) residues, but also that these cysteines locate at certain distances from one another and with the right stereochemistry. We note, however, that ferredoxin-like domains that contain iron–sulfur clusters are highly abundant and span 16 ECOD X-groups (and likely more X-groups not annotated as such). Despite that, shared themes are detected only between few of these X-groups. That not all of the iron sulfur–binding proteins share the same theme suggest a common evolutionary origin rather than convergence.

Supplementary figure 9S, Supplementary Material online, shows a theme shared by ECOD e3ephA1 and e4dgwA1 of the P-loop (2004) and alpha-bundle (3402) X-groups, respectively. In this case, the structures of the shared theme of 26 residues (with 50%/28% sequence similarity/identity) are very similar in both domains (C-alpha RMSD = 1.38 Å) and they wrap, or enclose, similarly positioned Zinc ions. Zinc binding in 4dgw (an SF3a core protein) is coordinated by four invariant residues: C282, C285, H298, and H304 (Lin and Xu 2012). The four equivalent positions in the variation in e3ephA1 feature the exact same amino acids. In this case too, ConSurf analysis indicates that both sequences are evolutionarily conserved, each within the context of its domain and so are the two cysteines and two histidines (assigned with the maximal ConSurf conservation grade). Structurally, this theme appears detached from the rest of the domain in both contexts, suggesting that it could be autonomously folded, perhaps stabilized by the bound ion. Thus, this theme may represent a case of cooption, namely of a segment taken from one protein being fused to another protein.

Finally, another tantalizing evolutionary link we have identified connects the two most ancient and diverse lineages of Rossmann-like and P-loop NTPases and is analyzed in detail separately (Longo et al. 2020). Similar to the above presented cases where the shared theme includes a key ligand binding motif, that is, iron–sulfur clusters, this shared theme includes two key functional elements—a phosphate-binding loop and an aspartate that binds the ligand’s ribose moiety in Rossmanns and the catalytic metal ion in P-loop NTPases.

## Discussion

Our systematic search for bridging themes, short homologous sequences shared between proteins that are assumed to have evolved independently, yielded 525 representative domain pairs, spanning 73 different folds, or ECOD X-groups. These themes have at least 20 residues, and the average percent sequence identity/similarity between their corresponding variations is high (30%/64%, respectively), strongly indicating that these variations descended from a common ancestor. Organizing the representative examples as an overview network manifests the regions of the protein universe that they traverse. We find that the “alpha + beta” (22 instances) are the most common, followed in descending order by “all alpha” X-groups (20 instances), “all-beta” (13), “others” (11), and finally the “alpha/beta” (6) and “mixed alpha + beta and alpha/beta” X-groups (1). There are connections among X-groups of the same architecture (or A-class) (Levitt and Chothia 1976), but also many that cross class boundaries and involving all class combinations.

The largest connected component in our overview network includes (among others) themes shared between five alpha/beta X-groups of fundamental importance: flavodoxin-like, TIM-barrel, P-loop NTPase, Rossman-like, and Rossmann-like w/crossover. These groups share few notable features. They comprise the most ancient enzyme classes, and their founding function was phospho-ligand binding, and specifically binding of phosphorylated ribonucleotide ligands. Further, they bind the phosphate moiety at the N-terminus of a helix, and, with the exception of TIM-barrels, this helix always comprises the first helix (the binding element is usually described as a P-loop and resides between the first beta-strand and first helix) (Longo et al. 2020). Evolutionary linkages between some of these groups have been suggested. However, so far, only the link between TIM-barrels and flavodoxins has been established (Farías-Rico et al. 2014). Here, tangible links between all these folds are unraveled. The link between P-loop NTPase and Rossmann is of particular interest and is discussed in detail in an accompanying manuscript (Longo et al. 2020).

The alpha/beta X-groups are interesting because they include many superfamilies (Orengo et al. 1994), with various functions (Friedberg 2006; Osadchy and Kolodny 2011; Tóth-Petróczy and Tawfik 2014), and because they include domains that are considered ancient (Winstanley et al. 2005; Choi and Kim 2006; Caetano-Anollés et al. 2007; Ma et al. 2008; Wang et al. 2006, 2011; Edwards et al. 2013; Alva et al. 2015). There are other domain groups that are considered ancient in our overview network, for example, the cradle-loop barrel and the HTH (Wang et al. 2006, 2011). It was difficult to predict beforehand if the search for similar amino acid segments in protein space may reveal themes shared among ancient proteins because the signal for common origin, namely sequence homology, is expected to diminish over time. However, this could be balanced by 1) longer evolutionary time, which allows for more opportunities for the emergence of new folds by cooption of bridging themes, and 2) evolutionary pressure to preserve those segments that mediate key functions, rendering these also easier to detect. That we detected multiple shared themes among groups of likely ancient domains indicate that there may be cases in which these two effects dominate.

Our study complements the seminal study of Alva et al. who looked for ancient fragments in current-day proteins and identified a set of 40 short fragments that exist in different structural contexts (Alva et al. 2015). We address a closely related aim, relying on the ECOD classification (rather than SCOP used by them) and focusing on identifying pairs of seemingly unrelated domains with a shared theme. To compare the results of these two efforts, we analyzed Alva et al.’s fragments in a framework like ours. Comparing the distributions of the lengths of the shared segments and percent sequence similarity/identity, we see that the criteria for concluding that two protein segments have a common evolutionary origin are similar, but we tend to be more conservative with higher percent sequence identify/similarity and longer shared protein segments. Note, however, that in our data set, we did not require that the sequence-similar variations also have similar structures, and indeed, the structures are often different. Both studies rely on the state-of-the-art HHSearch sequence search engine (Soding 2005), but the search strategies differ. Our study employs our previously curated data set of themes (Nepomnyachiy et al. 2017) as “baits,” which we use to search for cases where variations of these themes appear in two or more different sequence and structural contexts. Using bait themes allows our search to “fish” sets of domains that are promising candidates, and even more specifically, to identify within the domains in these sets the evolutionary meaningful recurring segments, that is, the shared themes. That a variation of the shared segments must be first detected as a theme of at least 30 residues (and with our thresholds), appears as a limitation of the method. Indeed, many of the cases that Alva et al. report and that we miss are due to this. However, we believe that this added requirement focuses the alignment procedure on identifying evolutionary relevant cases. We expect that an ancient or bridging segment will be reused in protein space, and thus we also expect that it be detected as a theme.

The instances that we and Alva et al. (2015) detect differ markedly. Only 17 ECOD X-groups and 3 specific pairs of X-groups that share the same theme, appear in both sets (out of the combined 100 ECOD X-groups). The cases in the Alva et al. data set that we miss are generally due to our additional requirements, for example, minimal length, or that there is a matching bait theme. Nonetheless, in different regions of the protein universe, we find recurring themes of similar characteristics. In other words, even using our conservative thresholds, our approach significantly expands the set of documented events of protein segments shared between domains that are considered evolutionarily unrelated. Of particular interest are additional themes that include the ancient and diverse alpha/beta X-groups. The high-sequence similarity among the variations of these shared themes suggests that they have emerged from a common ancestor even though they are found in two contemporary domains that do not share an evolutionary origin in their entirety.

There are alternative explanations to protein segments in distinct domains that are similar to one another. Either they formed independently, and their similarity is due to pure chance or convergent evolution, or, as we try to argue here, they share common ancestry. It is hard to discern which is correct. If we can estimate the probability of forming these segments, and it is very low, we have a probabilistic argument that undermines convergent evolution. The lower the probabilities, the stronger the argument, for example, when the segments are long, or enriched with rare amino acids. Here, we use *E* values estimates of the state-of-the-art method HHSearch (Soding 2005) to identify segments for which we have probabilistic support that they are related to the same bait theme. Also, we kept only alignments that are relatively long and with significant *P* values, as these have additional probabilistic support. However, the probabilistic model is based on sequence and ignores possible dependencies that may be due to structure. Biophysical constraints, for example, due to the polypeptide backbone and protein structure, significantly limit structure space (Finkelstein and Ptitsyn 1987; Orengo et al. 2001; Skolnick et al. 2014), presumably also constraining sequence space. Independently formed proteins may converge to similar structures (Cheng et al. 2008) and thereby also to similar sequences (Murzin 1998), leading to false-positive hits. Being aware of these issues, we use the *E* and *P* values to identify interesting cases, rather than as statistical estimates. In this context, it is noteworthy that because this study involves many sequence comparisons one might wonder whether correction for multiple hypotheses testing is required. This would have been the case had we relied on the *E* and *P* values for statistical estimates. However, because we use them only as filter thresholds, the issue is moot. In summary, although it is safe to assume that most of the similarities detected are real, we cannot commit to each individual case.

For cases of shared ancestry, we now speculate about the turn of events that could have led to such current-day patterns. One possibility is that in the ancient past, a short ancestral theme existed on its own, that is, without the segments that flank it in the intact, contemporary domain, perhaps bound to an ion or mineral (e.g., figs. 6 and 7 and supplementary fig. 2S, Supplementary Material online, and Eck and Dayhoff 1966), a nucleotide (Narunsky et al. 2020), or RNA (Alva and Lupas 2018; Lupas et al. 2001). Over time, it may have duplicated, the two copies diverged (although not beyond the level of detection), and protein segments accumulated before and/or after both variations. Because sequence expansion happened postduplication, the two duplicates expanded independently of each other, leading to two different sequence contexts and often to two different folds. Furthermore, because the ancestral short theme is only a small part of the two otherwise different domains, the resulting overall structures are different, appearing today as two (or more) unrelated ECOD X-groups. The different context may in itself result in the very same sequence (theme) adopting a different structure (Kosloff and Kolodny 2008; Yadid et al. 2010; Lella and Mahalakshmi 2017; Dishman and Volkman 2018), and indeed, the structures of theme variations often vary, including sometimes even different secondary structures (figs. 3 and 7).

An alternative scenario is that the theme existed within the context of a functioning protein domain. Then, this part of the domain (i.e., that theme) was coopted, duplicated, and inserted into another protein domain (akin to copy-pasting), or alternatively, duplicated and fused to generate a repeat protein. That a protein segment can be grafted into another protein is supported by the protein design experiments which carried out such scenarios, including cases when the source and destination domains are of a different ECOD X-group classification (Eisenbeis et al. 2012; Farías-Rico et al. 2014). For themes that arose via the first scenario, one can deduce that they are ancient, and that their context (i.e., their flanking sequence segments) evolved to accommodate and extend these enclosed themes. In contrast, in themes that arose via the second scenario, selection would act to readjust the coopted theme to the new context. We currently cannot determine which (if any) of the identified themes followed the first scenario, and which (if any) was subject to the second one. Moreover, these scenarios are not mutually exclusive. That is, an ancient theme that acquired additional protein segments over time may have been subsequently grafted onto yet another protein. Phylogenetic reconstruction may shed light on this question. Regardless of the evolutionary scenario, that the themes are shared between domains from different X-groups attests for their evolutionary plasticity.

The feasibility of these scenarios can be examined experimentally. To this end, the themes that appear to mediate specific functions would be most convenient. Showing experimentally that an isolated theme retains its function (e.g., metal, iron–sulfur, or cofactor binding), even if at low level (e.g., weak affinity), would support the feasibility of the first scenario. On the other hand, grafting a theme from one context to a domain in another context would attest to the feasibility of the second scenario. A copy-paste event is a plausible explanation to the observed themes shared between the flavodoxin domains, e1ccwA1 and e4r3uC1 and the TIM-barrel domain, e1tqjA1, as supported by the experiments of Höcker and coworkers (Bharat et al. 2008; Eisenbeis et al. 2012; Farías-Rico et al. 2014) (even though the domains e1ccwA1, e4r3uC1, and e1tqjA1 were not studied directly in this experiment). To take a step further, a variation of the zinc-binding theme described in supplementary figure 2S, Supplementary Material online, could be experimentally inserted into a designed protein to introduce zinc-dependent regulation, or similarly, the iron–sulfur cluster-binding themes outlined in figure 6 or 7 could be added to a protein to endow binding of an iron–sulfur cluster ligand. In other words, the themes, and specifically, their reconstructed ancestral sequences, may be good candidates for protein engineering (in contrast with the contemporary variations that may have lost the contextual agility; Smock et al. 2016; Longo et al. 2020). Themes with ligand-binding function are particularly attractive candidates (Romero et al. 2018; Narunsky et al. 2020).

## Prospect

Domains are considered central to protein evolution (Jensen 1976; Soskine and Tawfik 2010; Kessel and Ben-Tal 2018). In single domain proteins, a primordial ancestor with promiscuous enzymatic activity could be the progenitor of a diverse family of proteins with various activities toward a multitude of substrates. For example, all contemporary TIM-barrel and Rossmann domains can be traced back to their respective common ancestors (Aravind et al. 2002; Caetano-Anollés et al. 2007; Laurino et al. 2016). Further, a contemporary multidomain protein with a novel function may have evolved by concatenation of primordial domains with their respective functions (Aravind et al. 2002; Chothia 2003; Vogel et al. 2004; Kessel and Ben-Tal 2018). However, it is yet to be known how the domains themselves evolved from smaller protein segments (Alva and Lupas 2018). Bridging themes may provide hints to this end, and some of them may report the building blocks from which today’s intact domains evolved. To fulfill this role, they would probably have to be at least marginally stable and should provide some advantageous biochemical function to be evolutionarily selected over other peptides. The iron–sulfur cluster-binding themes of figures 6 and 7 are good candidates in both respects. Iron–sulfur clusters are ancient relatively stable minerals (Raanan et al. 2020), which provide diverse stereochemistry and catalytic opportunities (Eck and Dayhoff 1966). Thus, an iron–sulfur cluster might have been a nucleus that bound amino acids and/or di- or tri-peptides that eventually elongated toward the emergence of an iron–sulfur cluster-binding theme, as hypothesized for ferredoxin (Eck and Dayhoff 1966; Mutter et al. 2019). In support of this hypothesis, the iron–sulfur cluster of the D dimerization subunit of the RNA polymerase (2pmz, fig. 5) contributes to stability, and mutations of the four cysteine residues that mediate its binding lead to aggregation of the subunit (Hirata et al. 2008). By analogy to domains, variations of a theme could have presumably emerged from a common ancestor, which then gave rise to different functions. It has been recently proposed, for example, that a theme comprising a beta-alpha-beta element may have given rise to both the Rossmann lineage (by virtue of binding FAD or NAD) and the ferredoxin one (by binding an iron–sulfur cluster, Raanan et al. 2020). That variations of the theme of figure 6 bind both F3S and SF4 iron–sulfur clusters supports this hypothetical scenario. To push the domains analogy even further, just as multidomain proteins may emerge by mix and match of existing domains (Chothia 2003), domains might emerge by mix and match of two or more preexisting themes. Protein engineering experiments could be conducted to examine function alterations within a given theme as well as concatenation of themes to integrate several functions and yield intact domains.

## Materials and Methods

The search carried out in this study is challenging because we look for domain pairs that satisfy two somewhat opposing criteria: 1) share short-matching segments of similar sequences, and yet are 2) dissimilar in their overall sequence and structure. To tackle this challenge, we search the ECOD database using a set of previously curated themes (Nepomnyachiy et al. 2017) as baits and look for themes that appear in two or more dissimilar domains. Themes are subdomain recurring protein segments that we have previously identified from all-vs-all sequence alignments of (non redundant set of) PDB proteins (Nepomnyachiy et al. 2017). More specifically, we used HHblits (Remmert et al. 2012) to calculate a hidden Makov model (HMM) for each recurring segment (adding sequences from uniclust30). The themes in the precurated data set are these HMMs of the reused segments. The lengths of the themes in this curated set vary but are at least 30 residues. Relying on the preprocessing step of curating the set of themes, and using these as baits holds two advantages: 1) it restricts the search, and rather than considering all domain pairs, it focuses on the pairs that have a segment similar to the same theme and 2) we can derive the matching short segments within these domains from the parts aligned to the bait theme. The advantages of this search method come hand-in-hand with its inherent limitation: shared parts must first be detected as a theme (of at least 30 residues). Our bridging themes were identified using a total of 261 bait themes. Although most (136) baits identified exactly two domains (one in each X-group), some identified more than two domains. Supplementary figure 10S, Supplementary Material online shows the histogram of the number of domains identified by our baits.

We used HHSearch (version 3.0.0) to compare a set of 12,681 previously curated themes to a 70% NR set of ECOD (43,830 domains in version develop210 and 43,281 in the updated develop263). We used the *E* value threshold of 10^−3^ and coverage = 0.85 to identify significant hits and find 22,381 ECODs that are similar to any of the themes (spanning 3,137 F-groups, 746 H-groups, and 646 X-groups). About 1,698 of our themes are similar to ECODs that are in different X-groups. Because there is extensive redundancy among the themes, we next identify representative meaningful examples. For each such theme, and for each pair of X-groups, X_1_ and X_2_ that it matches, we consider all *n*_1_ domains it matches in X_1_ and all *n*_2_ domains that it matches in X_2_; we consider the *n*_1_×*n*_2_ potential domain pairs. For each domain pair, we calculate the SW alignments of the parts before and after, and the SW or NW alignment of the recurring part that matches the theme. When the local SW aligner finds an alignment of the recurring parts that matches more than 20 residues, we opt for this alignment. Because the local SW aligner may discard the beginning and the end of a segment if they do not improve the overall alignment score, it may result in alignments that match fewer yet more similar residues and, specifically, fewer than 20 residues. In the 104 such cases (∼20%), we consider the NW alignment. If *n*_1_×*n*_2_ equals 1, then we return this example. Otherwise, we normalize the scores of the matching recurring parts and the parts before/after (by subtracting the mean score and dividing by the standard deviation) and identify the two examples with the maximal difference between the (high) alignment score for the recurring part and the (low) alignment score for the parts before/after. For every pair of domains, if they share more than one recurring theme (regardless of the similarity among the themes that identified these domains), only the longest theme was kept.

Finally, for each aligned pair of protein segments, we calculate the properties of the alignment: the number of aligned residues, the percent identity, the percent similarity (using Blosum62), structural similarity measures, and the *P* value. The *P* value measures the significance of the alignment score with respect to scores of alignments of random segments (drawn from the same distribution). We estimate the parameters of their extreme value distribution from the scores of the alignments between the first segment and 1,000 randomly chosen segments drawn from a multinomial distribution estimated from the second segment. We calculated the following structural similarity measures: 1) RMSD of the C-alpha atoms of the aligned residues after optimal superpositioning, 2) measures of structural similarity after structurally aligning the matching domain segments with TM-align (Zhang and Skolnick 2005): TM score and RMSD of the structurally aligned residues, 3) dRMSD (distance RMSD) of aligned residues, 4) percent agreement of secondary structure assignment. We derived the secondary structure assignments from precalculated DSSP files (Kabsch and Sander 1983; Touw et al. 2015) (chains 1we3, 3iyg, 3k1q, 4b4t, and 4di7 are unavailable). (5) Percent change in contact maps of aligned residues. We follow the CASP convention (Schaarschmidt et al. 2018) by which two residues are in contact if the Euclidian distance between their C-beta atoms (C-alpha in the case of Glycine) is below a threshold; we used a 9 and 11 Å as thresholds. The percent similarity is the percent of differing entries in the 0/1 lxl matrices representing the contact maps (where l is the number of aligned residues), that is, the number of differing entries/$l^{2}$.

To identify the domains with binding residues within 4.5 Å of a ligand, we collected 129 relevant ligand codes. To identify these, we started with all ligand codes in the resNames field of the HETATMs and removed crystallographic additives (listed in Drwal et al. 2017), ligands that do not appear in the BioLiP frequency file, modified residues (e.g., MSE, CME), HOH, UNL, and UNX. About 288 of the domains include one of these ligands, and we identified all residues with an atom within the 4.5 Å distance from it as binding it. BioLiP lists binding residues in most (217) of these domains. The number of binding residues found by the two methods is very similar (0.86 correlation).

The data are organized as two Cytoscape (Saito et al. 2012)/CytoStruct (Nepomnyachiy et al. 2015) sessions: one for the overview network, and one with all the nested networks. The colors of the nodes are based on their ECOD A-group classification, grouped into structural classes (Levitt and Chothia 1976): all-α in blue (α-arrays, α-bundles, α-complex topology, α-superhelices), all-β in red (β-barrels, β-complex topology, β duplicates or obligate multimers, β-sandwiches), α + β in yellow (α + β complex topology, α + β duplicate or obligate multimers, α + β three-layers, α + β two layers), α/β in green (α/β barrels, α/β three-layered sandwiches), mixed α + β and α/β in yellow-green, and others in cyan (extended segments, few secondary structure elements). In the downloadable session, a right-click on a nested-network edge opens 1) PyMOL (Schrodinger 2010) to show the structures of the two domains, with the themes highlighted and superimposed on each other or 2) BioEdit (Hall 1999) to show the aligned sequences. In the online version, a click on the edge opens the nested network, in which one can click on edge to download the PyMOL script or shift + rleft-click to see the superimposed structures and the aligned sequences in the web browser.

### Comparison with the Set of Alva et al. (2015)

We downloaded the set from the supplementary to figure 3 in Alva et al. (2015). For each of the segments in the MSAs of their set of ancient fragments, we identified the ECOD of that segment and recorded its ECOD classification. In two cases, we shortened the segment by one residue because it fell on a domain boundary (1NT0 146-163, 1JX4 177-198). We focus on pairs of sequence segments that are in the same fragment, that is, are aligned to each other in the MSA of that fragment, and that the ECOD X-groups of the domains of these segments differ; there are 286 such cases. We compared the properties of the two sets and their overview networks (supplementary figs. 8S and 9S, Supplementary Material online).

The two efforts overlap in 17 ECOD X-groups, and three links between X-groups. The themes in the shared links appear similar. The first link connects X-groups 101 and 819 (fragment #1 in Alva et al.). Both sets include the domain e1nr3A1 (X-group 819) and even the same residues (7-27 in our data set, 8-27 in theirs). In the X-group 101, both data sets include a domain from the 101.1.4.66 F-group (e1r69A1 in our set vs. e2r1jL1 in theirs). The second link connects X-groups 187 and 205 (fragment #18). There are many cases of domains from the same F-groups: 187.1.1.5 (4 domains in each set) and 205.1.1.8/12/93 (8 domains in ours vs. 6 in theirs). The third connects X-groups 604 and 109 (fragment #28). The domains in both sets are from the F-group 604.12.1.4, and in the other X-groups, the domains only have the same ECOD T-group 109.4.1. As for the X-groups that although found in both efforts are linked to different X-groups: the domains are not necessarily similar. For example, in the 2003 X-group (Rossmann-like), Alva et al.’s set includes domains from the T-groups 2003.1 (Eck and Dayhoff 1966; Grishin 2001; Aravind et al. 2002; Dokholyan et al., 2002; Greene et al. 2007; Cheng et al. 2014; Edwards and Deane 2015; Alva and Lupas 2018) (fragment #8), whereas all domains in our set are from 2003.1.1; in the one X-group (cradle loop barrels) only the X-group is the same: Our domain is from 1.2.1.17, whereas theirs (fragment #15) are from a different group: 1.1.5.147.

Cases found in the Alva et al. set, and not in ours, are due to different reasons. Some are trivially missing from our set because we use ECOD that is less conservative than SCOP (the domains in fragments (2, 6, 7, 9, 11–14, 16, 19, 21, 22, 26, 27, 33, 34, 37) have the same ECOD X-group classification), or because of our more conservative thresholds: fragments (4, 15, 17–18, 20, 23, 25–26, 37, 40) are shorter than 20 residues. In fragments (3, 8, 28–32, 35–36, 38–39), our precurated set of bait themes does not include themes that are from the domains of one of the folds for that fragment (implying that we cannot find cross X-group similarities for this case). That there is no match to any segment in specific domains to any of our bait themes, may be due to these domains removed from our 70% NR set of ECOD. Hence, if a particular domain is not in our ECOD set of domains, we checked if any of its close homologues (namely, all the domains with the same ECOD A.X.H.T.F classification) was matched to a bait theme. Finally, the domains in fragments (8, 10, 24), while matched to some bait themes, with sufficiently low *E* values, it was not the same bait themes. In summary, although both approaches are based on the HHSearch engine, the significant methodological differences lead to different results.

## Supplementary Material

Supplementary data are available at *Molecular Biology and Evolution* online.

## Supplementary Material

msab017_Supplementary_Data
