# Erratum to: Bridging themes: short protein segments found in different architectures

**DOI:** 10.1093/molbev/msab097

**Published:** 2021-05-03

**Authors:** Rachel Kolodny, Sergey Nepomnyachiy, Dan S Tawfik, Nir Ben-Tal

*Molecular Biology and Evolution*, msab017, https://doi.org/10.1093/molbev/msab017

The alpha and beta symbols were erroneously missing from figures 4 and 5. This has now been corrected online.

