# Supplementary material for: Bridging Themes: Short Protein Segments Found in Different Architectures
Source: Mol Biol Evol. 2021 Jan 27;38(6):2191–208. doi: 10.1093/molbev/msab017 (PMC8136508; doi:10.1093/molbev/msab017)
Supplement: msab017_Supplementary_Data [file msab017_supplementary_data.pdf]

Bridging themes: short protein segments found in different architectures

Rachel Kolodny[1,*], Sergey Nepomnyachiy[1], Dan S. Tawfik[2], Nir Ben-Tal[3,*]

[1]University of Haifa, Department of Computer Science, Haifa, Israel

[2]Weizmann Institute of Science, Department of Biomolecular Sciences, Rehovot, Israel

[3]Tel Aviv University, George S. Wise Faculty of Life Sciences, Department of Biochemistry and Molecular Biology, Tel Aviv, Israel

[*]Corresponding authors: trachel@cs.haifa.ac.il, bental@tauex.tau.ac.il

Supplementary Material

X-group id	#dataset domains in X-group	X-group name	Example domain	Architecture	#dataset domains In architecture
101	24	HTH	e1efaA1	α-arrays	46
103	4	RuvA-C	e2dhyA1		
108	3	EF-hand	e2omzA4		
138	2	NO_X_NAME	e3bgeA1		
148	1	Histone-like	e4xguA2		
187	5	alpha-helical ferredoxin-like	e1e7pB1		
3206	1	NO_X_NAME	e2kr0A2		
531	5	Domains of FliG	e1lkvX1		
589	1	NO_X_NAME	e3rfwA2		
150	4	Ferritin/Heme oxygenase/4-helical cytokines	e2cwlA1	α-bundles	15
3403	1	NO_X_NAME	e4dgwA1		
3763	1	NO_X_NAME	e3ezuA2		
3847	1	NO_X_NAME	e5c0sA1		
593	2	NO_X_NAME	e1iokA1		
601	1	Four-helical up-and-down bundle	e1ucuA4		
604	2	Spectrin repeat-like	e2v6yA1		
632	3	immunoglobulin/albumin-binding domain-like	e1l6xB1		
3601	1	NO_X_NAME	e2ev4A1	α-complex topology	1
109	63	Repetitive alpha hairpins	e1awcB1	α-superhelices	65
3238	2	Mitochondrial mTERF-like	e2ypfA1		
1	1	cradle loop barrel	e1ihmA2	β-barrels	19
2	2	OB-fold	e2wacA2		
236	4	GroES-like	e2dphA1		
4	6	SH3	e2diqA1		
6120	2	NO_X_NAME	e3zfoA1		
70	4	beta-clip	e3n71A1		
3702	4	NO_X_NAME	e1rp5A6	β-complex topology	4
207	5	Single-stranded right-handed beta-helix	e1dceA3	β-duplicates or obligate multimers	10
208	1	Single-stranded left-handed beta-helix	e3pr7B1		
3512	4	NO_X_NAME	e1p9hA3		
10	5	jelly-roll	e3eqeA1	β-sandwiches	10
11	1	Immunoglobulin-like beta-sandwich	e4r5oA2		
3414	1	NO_X_NAME	e4l3aB3		
3856	2	NO_X_NAME	e4oj6C1		
3857	1	NO_X_NAME	e1lktA1		
2002	3	TIM beta/alpha-barrel	e1tqjA1	α/β barrels	3
2003	64	Rossmann-like	e1ek6A1	α/β three-layered sandwiches	179
2004	36	P-loop domains-like	e1gkyA1		
2007	69	Flavodoxin-like	e1a04A2		
2487	5	The swivelling" beta/beta/alpha domains"	e1a6dB3		
7523	4	NO_X_NAME	e1eljA2		
7577	1	NO_X_NAME	e4nogB1		
206	1	NO_X_NAME	e4redA1	α+β complex topology	5
219	1	Cysteine proteinases-like	e3s0qA1		
4064	1	NO_X_NAME	e1ej6C1		
5093	2	NO_X_NAME	e3rkiA1		
3567	6	NO_X_NAME	e3k9aA1	α+β duplicates or obligate multimers	6
223	1	Profilin-like	e3kyqA1	α+β three layers	15
2485	13	Thioredoxin-like	e1z9hA2		
4031	1	NO_X_NAME	e3a5xA4		

205	16	NO_X_NAME	e1dwlA1	α+β two layers	62
214	1	NO_X_NAME	e2cr4A1		
221	24	beta-Grasp	e1p0rA1		
225	1	ATPase domain of HSP90 chaperone/DNA topoisomerase II/histidine kinase-like	e2q2eB1		
284	2	FKBP-like	e2jzvA1		
304	8	Alpha-beta plaits	e1b7fB2		
305	1	DCoH-like	e2pmzS1		
308	1	ClpS-like	e2z2lD1		
327	2	Alpha-lytic protease prodomain-like	e1yj7A2		
3953	1	NO_X_NAME	e3sluA3		
4305	1	NO_X_NAME	e2d9bA1		
6043	1	yfeY-like	e4ygtA2		
809	2	BLIP-like	e2km7A1		
819	1	NO_X_NAME	e1nr3A1		
2498	1	Zincin-like	e3j1bA2	mixed α+β and α/β	1
3100	3	NO_X_NAME	e2kogA1	Extended segments	7
3362	1	NO_X_NAME	e2b9bA3		
3462	1	NO_X_NAME	e3v6iX2		
3464	2	NO_X_NAME	e3j0jL2		
375	6	Rubredoxin-like	e1k83L1	few secondary structure elements	79
376	2	RING/U-box-like	e2ysmA2		
377	7	Glucocorticoid receptor-like	e1b8tA3		
386	55	beta-beta-alpha zinc fingers	e1meyC1		
389	1	EGF-like	e1igrA3		
904	2	B-box zinc-binding domain-like	e2didA1		
906	6	CCCH zinc finger	e1rgoA2		

Table 1S: The list of ECOD X-groups and the number of domains in each of the X-groups for the set of domains in the bridging themes dataset.

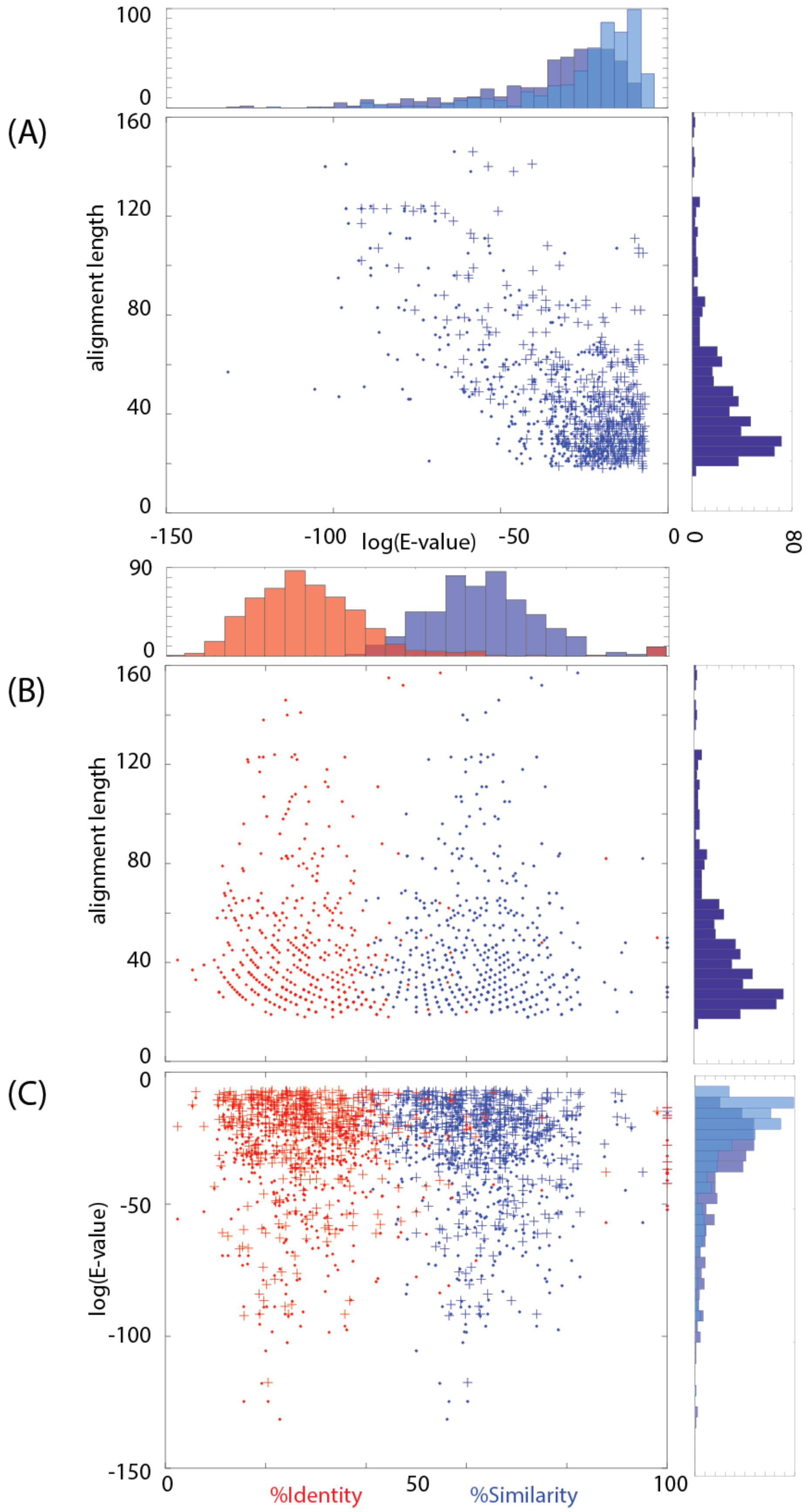

Figure 1S: Relating similarity measures:
(A) For the 525 pairs in our dataset, we plot the log(E-value) vs. the alignment length. For each pair of variations, there is a single alignment length value, but two E-values, reported by HHSearch for the “bait” theme and the two domains. We plot these with a ‘+’ (maximal) and ‘.’ (minimal) mark. The histograms of these values are shown to the right and on the top. As expected, longer alignments have lower E-values. (B) The sequence similarity/identify of alignments vs. the alignment length. (C) The sequence similarity/identity of alignments vs. the E-values. The similarity/identify are not correlated with the alignment lengths /E-values.

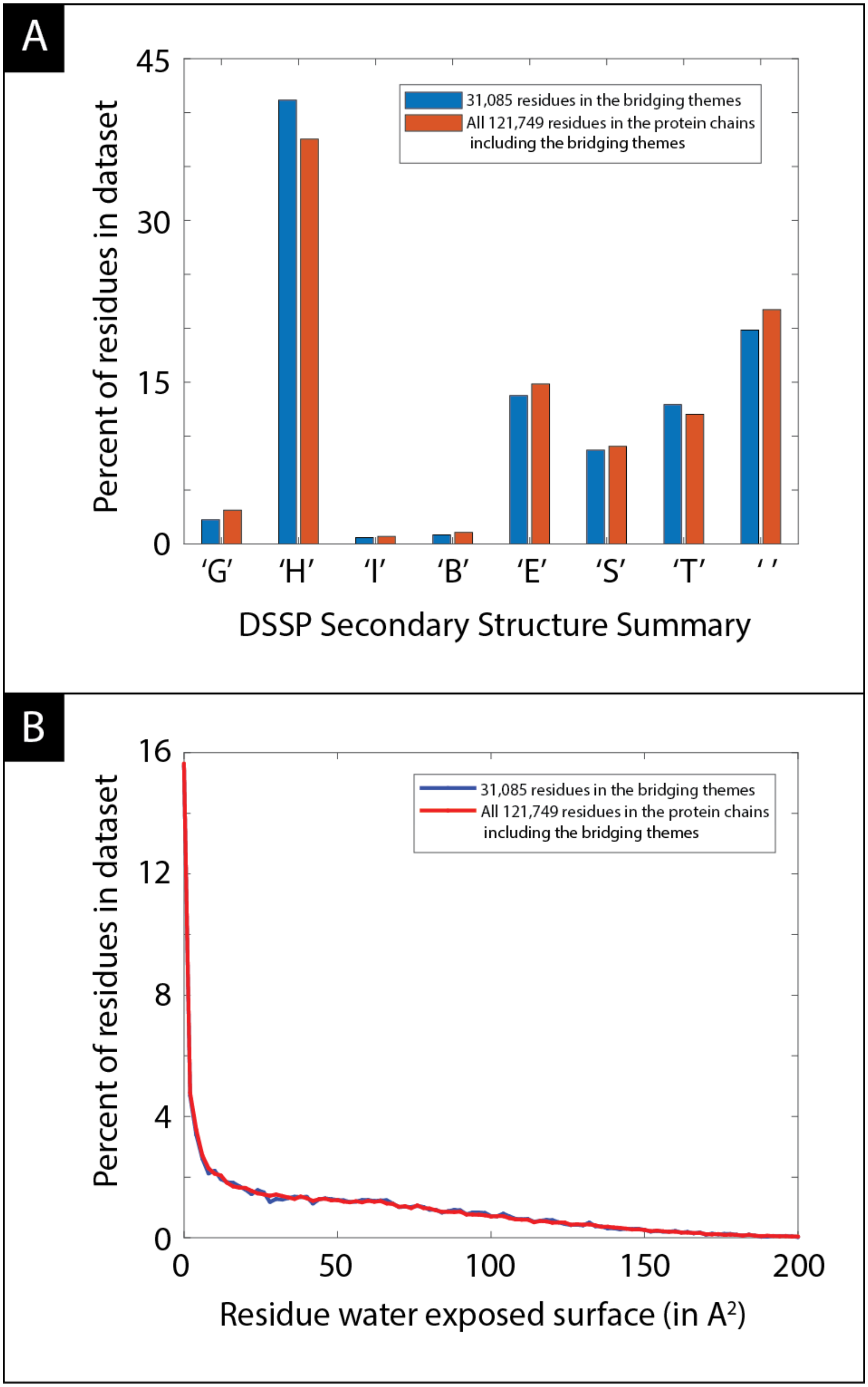

Figure 2S: The secondary structure assignments (panel A) and solvent accessibility (panel B) distributions of the residues in the bridging themes (blue) and all residues (red) are similar. The nomenclature and values are derived from DSSP (Kabsch & Sander, 83): G is 3_{10}-helix, H is α-helix, I is π-helix, B residue in isolated β-bridge, E is extended strand, S is bend, T is hydrogen bonded turn and a blank (the last column) is a loop or irregular.

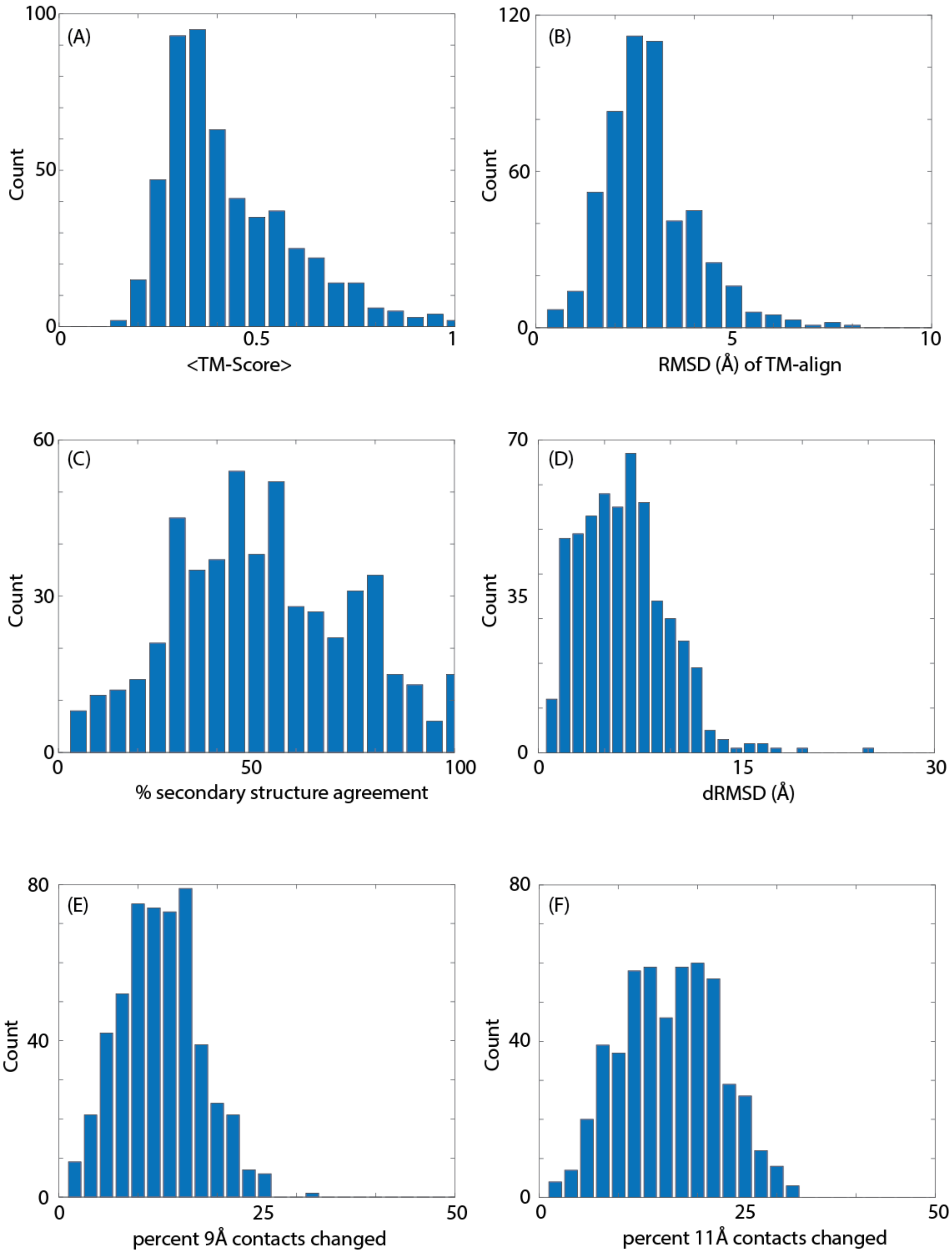

Figure 3S: Histograms of structural similarity measures comparing the variations of the bridging themes. The measures in Panel A,B are calculated by TM-align. TM-scores range between 0-1, with scores > 0.5 indicating the same fold (25% of the pairs), and <0.3 indicating random structural similarity (30% of the pairs). The RMSDs of the structurally aligned residues (panel B) are lower than those the sequence aligned residues (Figure 3 in the main document) because these are only a subset of the residues (and the ones that are structurally similar). Panel C shows the percent secondary structure agreement, and we see that in many cases, this agreement is not high. Panel D shows the dRMSD, Panels E,F show the percent contacts changed using two thresholds (9 Å and 11 Å).

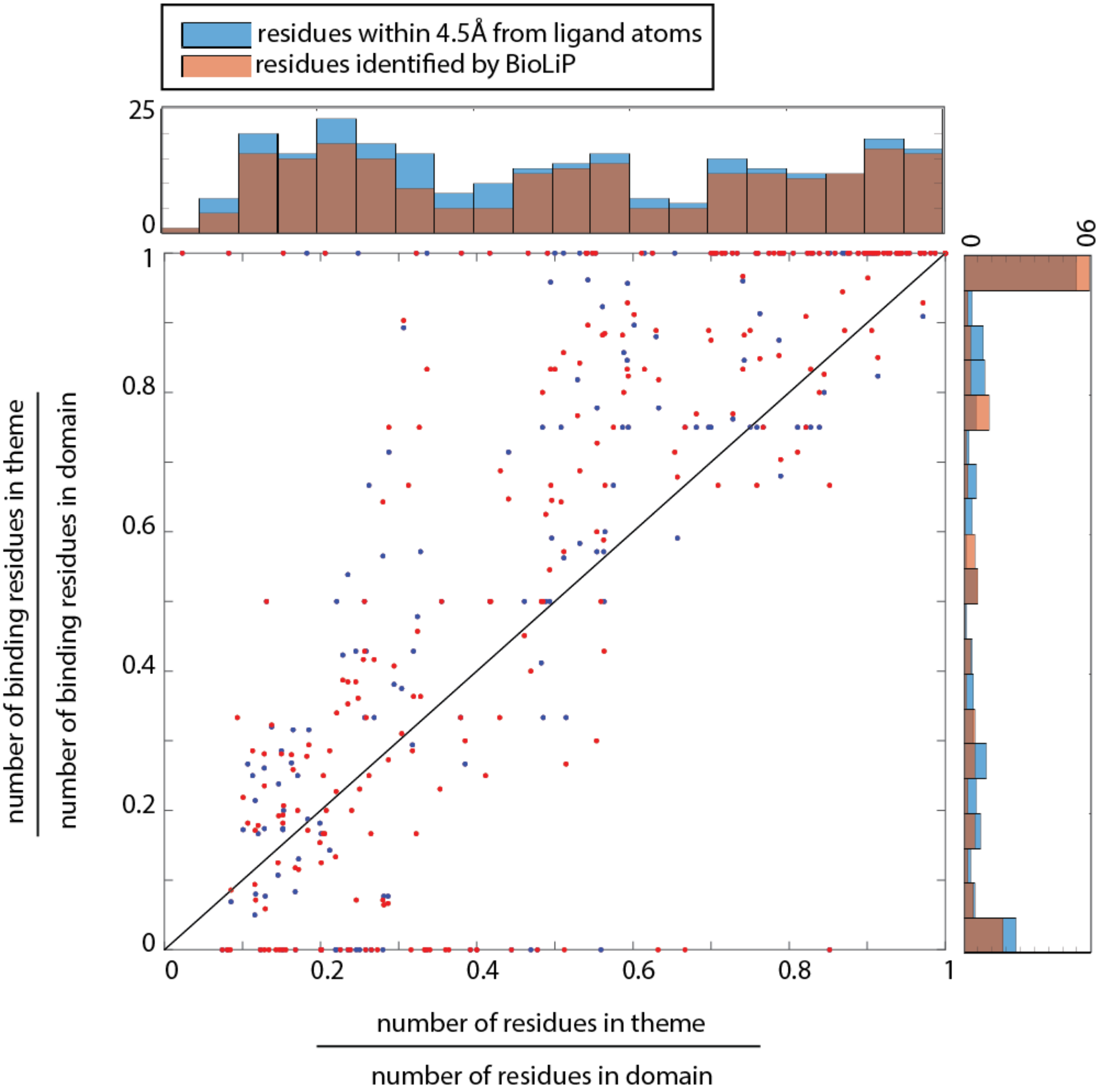

Figure 4S: Comparing two rations: along the x-axis, the ratio of the number of residues in theme to number of residues in domain vs. along the y-axis the ratio of the number of binding residues in theme to total number of binding residues in domain. T cumulative histograms of these ratios are also shown. The ratio along the x-axis serves as the background distribution of the percent of residues that are in a bridging theme for domains for which we have binding data: In blue we show the histogram for the 263 domains in which we identified binding of a ligand with 4.5 Å of one of its residues, and in red-brown, the dataset of 217 domains for which we have BioLiP data. In both datasets, we see an enrichment of the high ratios for binding residues, i.e., there are more cases in which binding residues are within a bridging theme (shown as datapoints to the upper-left of the diagonal line).

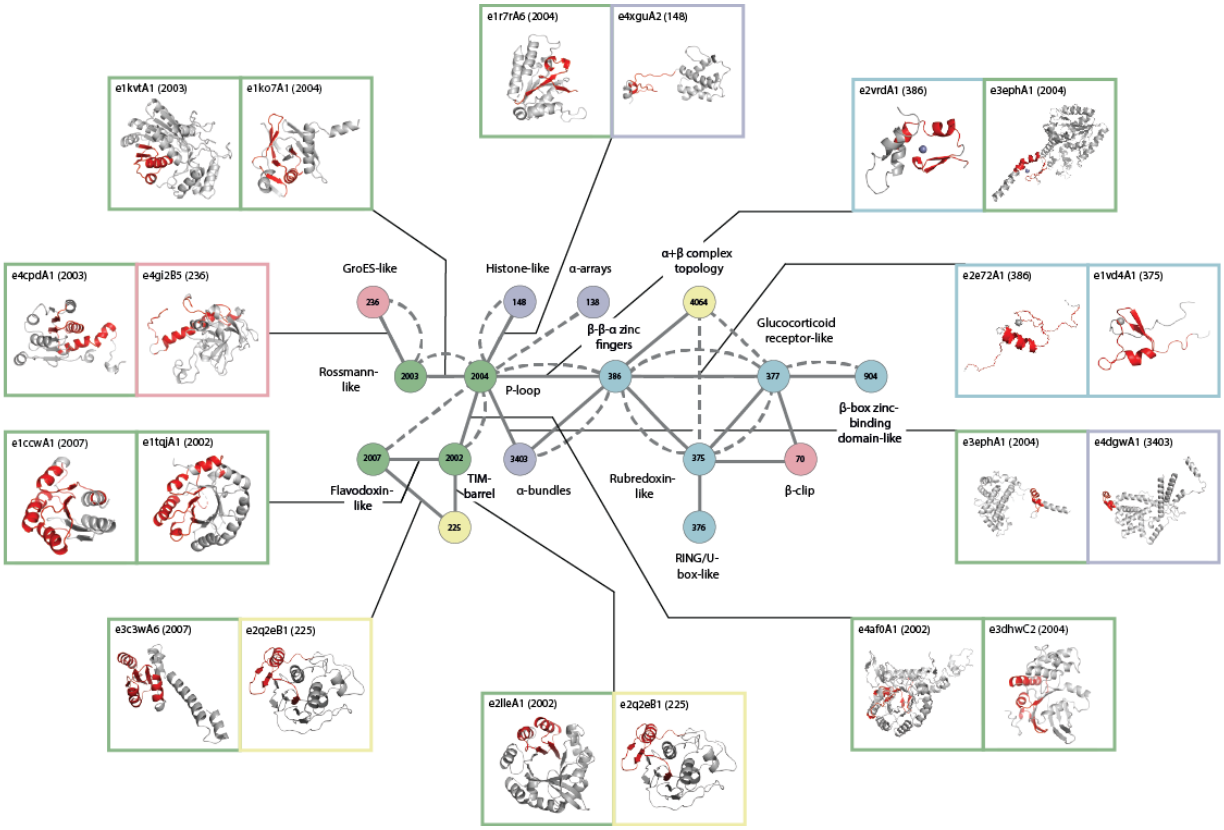

Figure 5S: A part of the largest connected component in the overview networks (Figure 4). An arbitrarily chosen example is shown for each pair of X-groups that share a theme. Shown are the structures of the two domains, colored in gray, and the shared theme highlighted in red. Many of the ECOD X-groups in this connected component are considered ancient, including the P-loops, the Rossmanns, and the TIM-barrels.

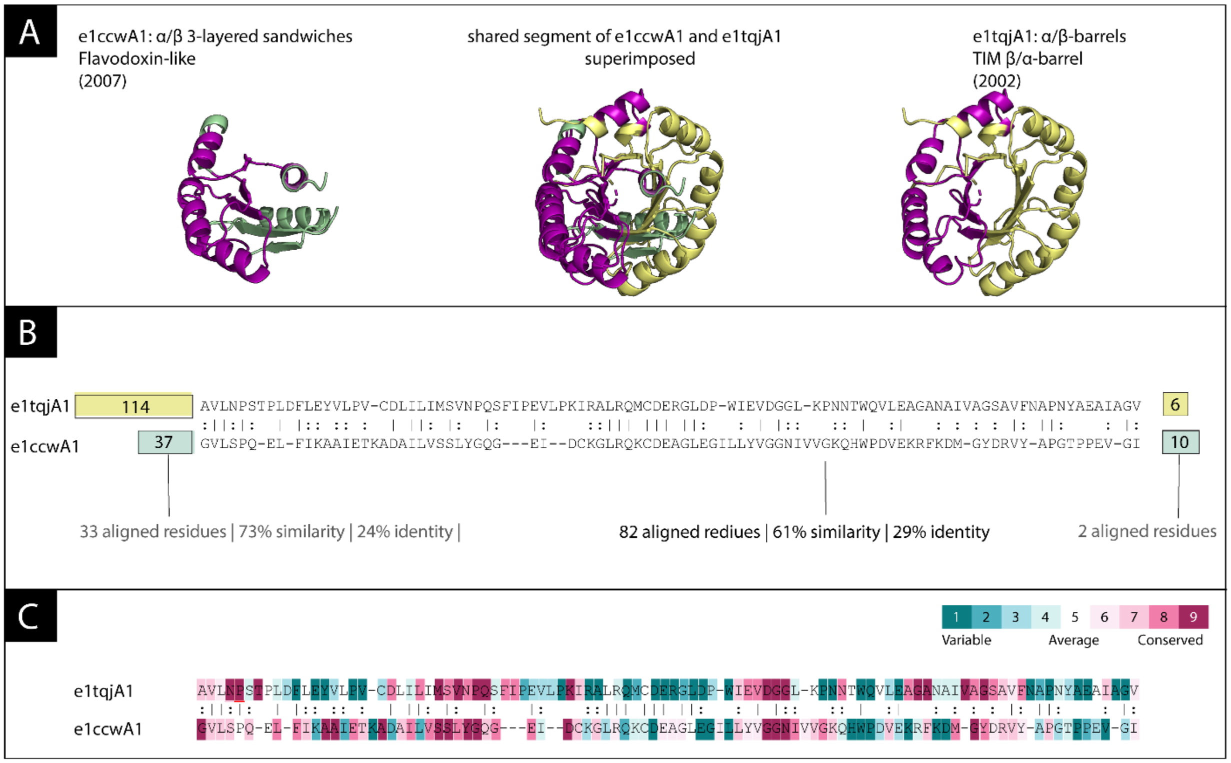

Figure 6S: A bridging theme shared between e1ccwA1 (Flavodoxin-like domain) and e1tqjA1 (TIM beta/alpha barrel) (A) The overall structures are characteristic of their folds: the structures are shown superimposed in the middle and separated for clarity. The shared theme is colored in violet. The structures of the variations are very similar (Cα RMSD = 1.38 Å) while the rest of the structures is not. (B) Sequence alignment shows that the similarity between the variations on the shared theme are similar to the respective N-terminal regions of the domains (C) ConSurf analysis of the corresponding proteins (1tqj and 1ccw) and their respective homologues.

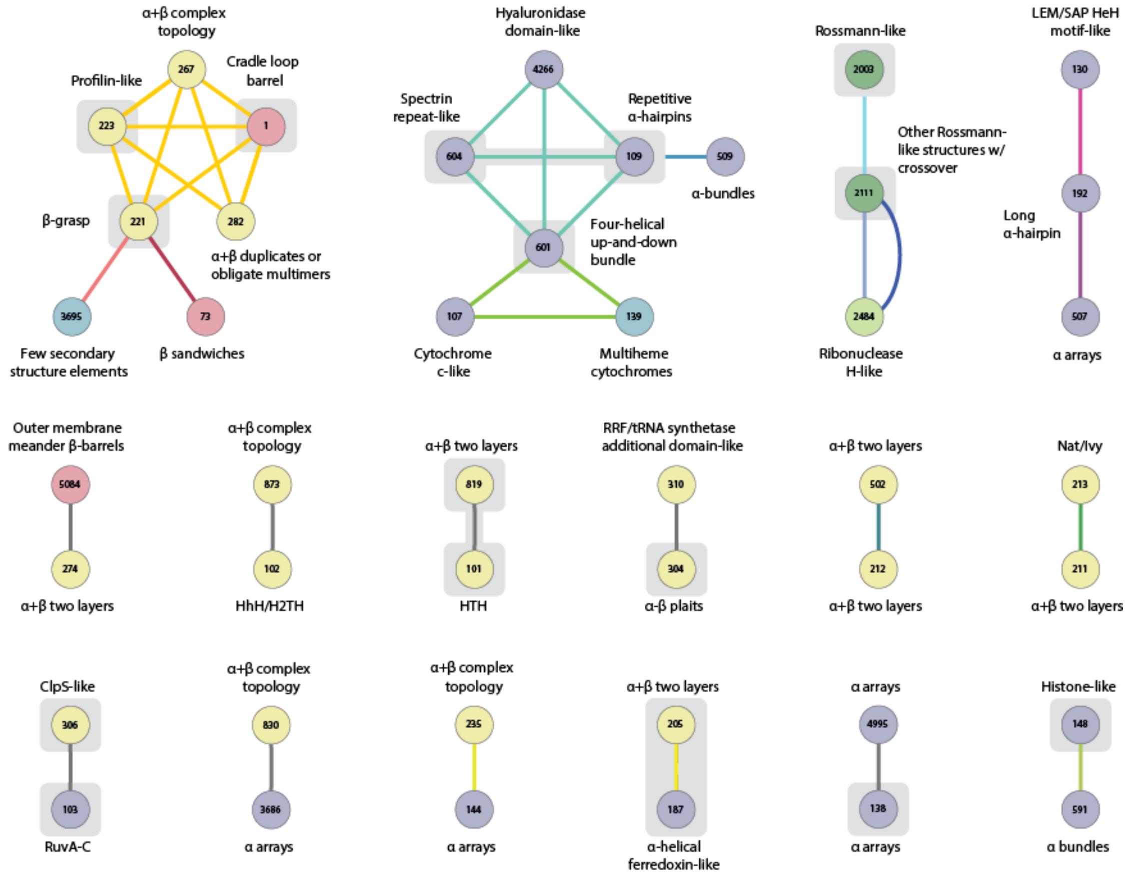

Figure 7S: An overview network for the Alva et. al. set [1]. We use Figure 4's color coding, and add the yellow-green shade for the mixed a+b and a/b class (2484). The color of the edge connecting two X-groups encodes the serial number of the fragment in the set: e.g., domains from X-groups 601, 107, and 139 appear in the same fragment (#17). The seventeen ECOD X-groups and three edges that were also found among our agile themes are shown with a gray background.

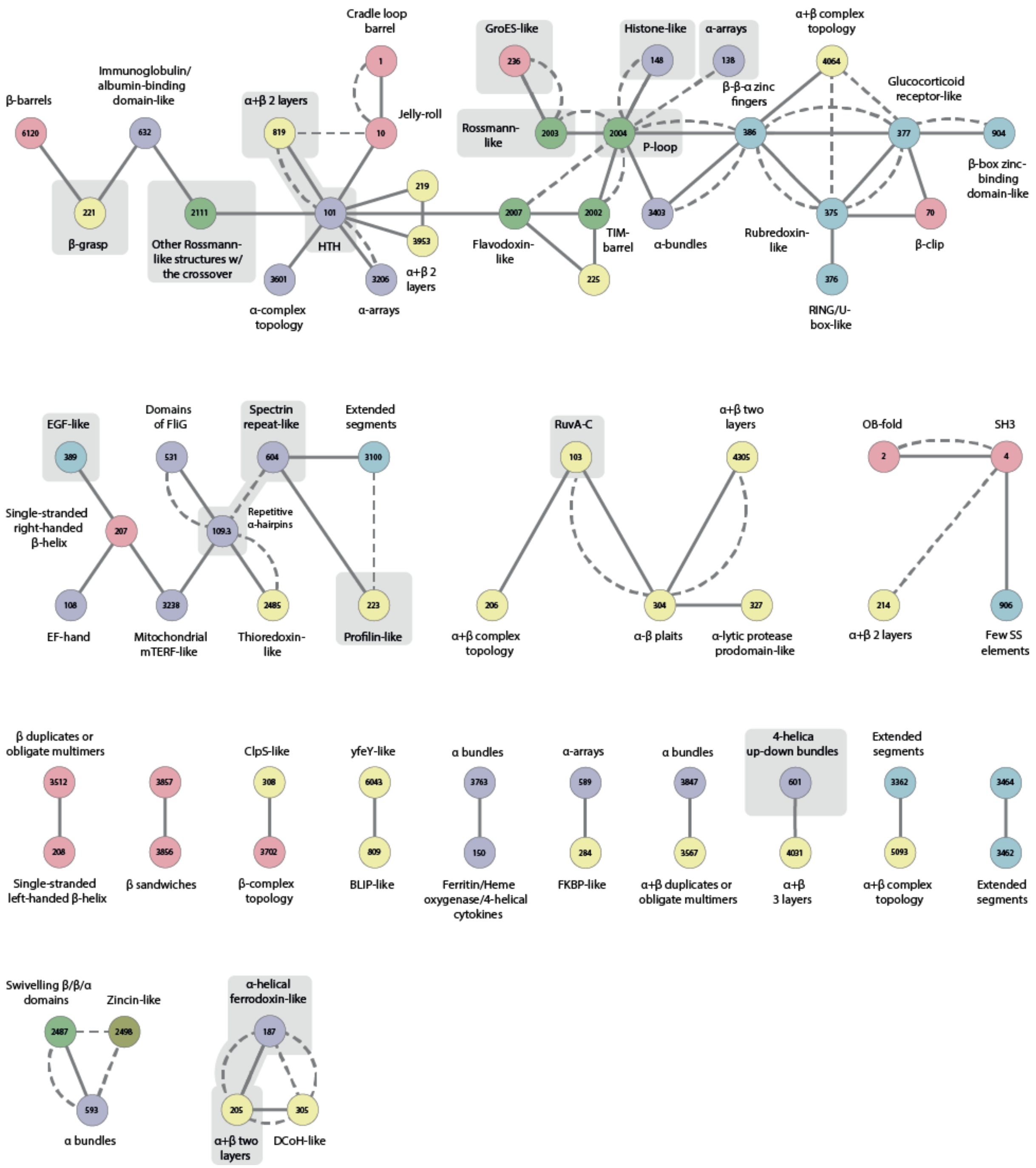

Figure 8S: Highlighting the regions in protein space with shared themes found by us and by Alva et al., within our overview network (see Fig. 4). There are 17 ECOD X-groups and 3 edges that were also found in Alva et al.'s dataset shown with a gray background. Node colors correspond to class of the X-group: blue for all-α, red for all-β, green for α/β, yellow for α+β, and cyan for others, and edges connect pairs of different X-groups for which we found a common theme in a domain in each of the two groups.

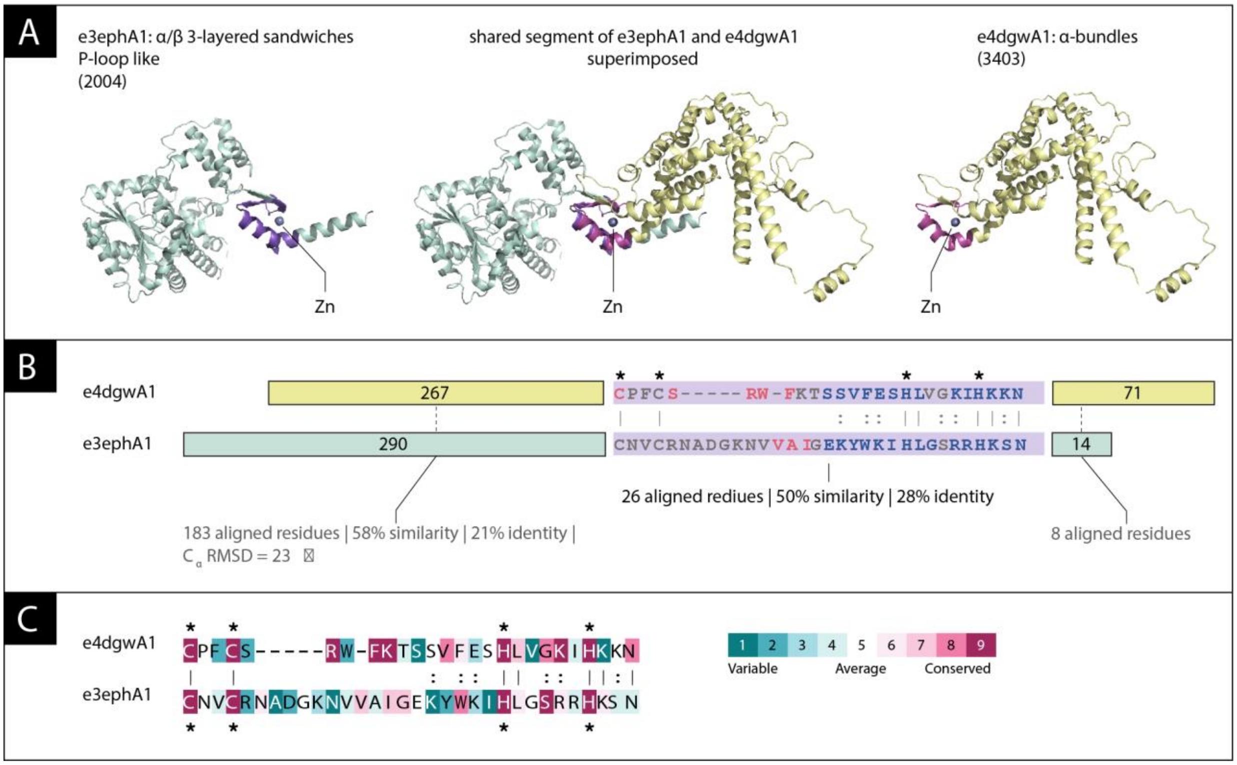

Figure 9S: A zinc-binding theme shared between the domain e3ephA1 from the 2004 X-group and the domain e4dgwA1 from the 3404 X-group. (A) The overall structures are different. Domain e3ephA1 (left) is a P-loop like domain from the alpha/beta A-group, and e4dgwA1 (right) is from the all-alpha domain-group. The 26 residues long shared theme (pink) binds zinc (grey sphere). When structurally superimposing the shared theme (middle), the structures of the two are very similar (Cα RMSD = 1.38 Å) while the rest of the structures is not. (B) Sequence alignment shows that the similarity between the variations on the shared theme are similar to the respective N-terminal regions of the domains, and much higher than the similarity of the respective C-terminal regions. The four residues that mediate zinc binding in both domains, marked with asterisks, align to each other in both variations. (C) ConSurf analysis of the corresponding proteins (4dgw and 3eph) and their respective homologues show that the zinc binding residues (marked with asterisks) are highly conserved.

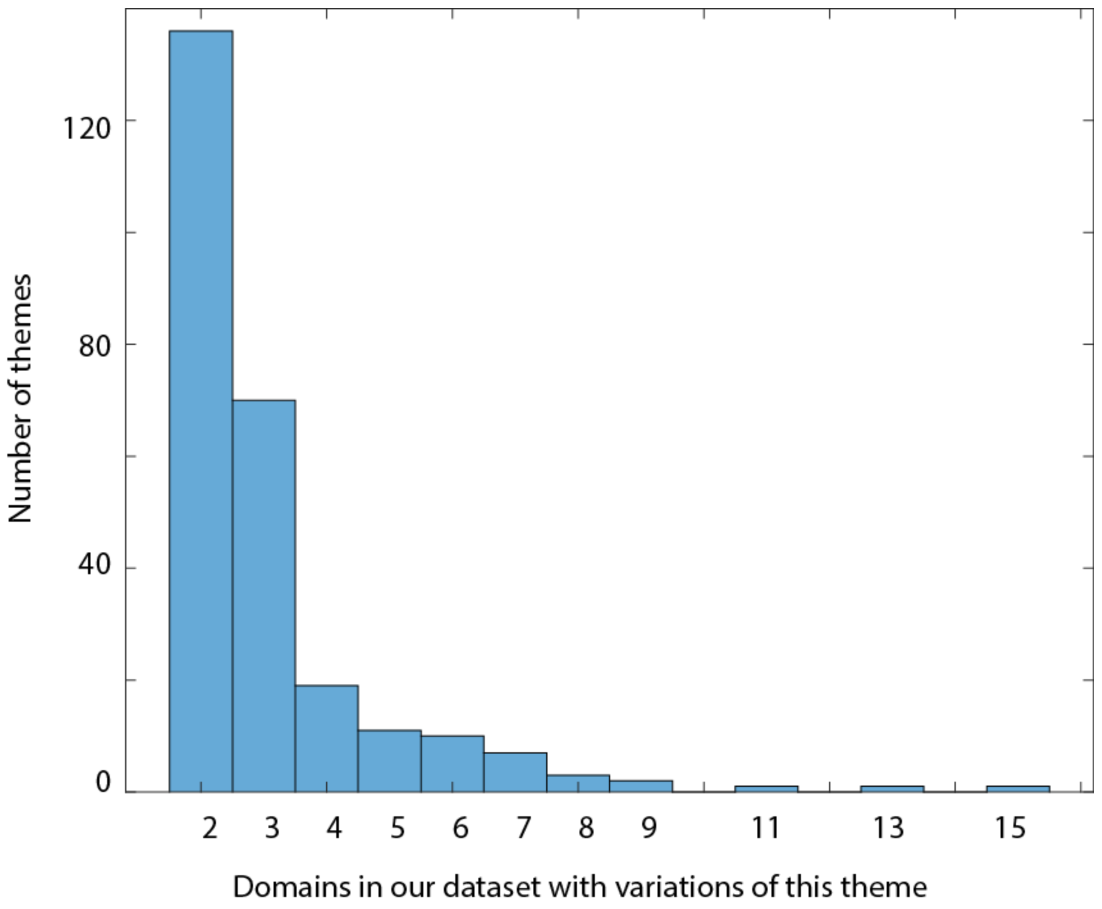

Figure 10S: The number of domains identified by each of the bait themes that contributed variations to our bridging data set. Altogether, 261 bait themes successfully identified bridging themes, and the number of domains that each identified varies: over half (136) identified only the two domains in different X-groups. The maximal number of domains related in our set by one bait theme is 15.

References

1. Alva, V., J. Söding, and A.N. Lupas, *A vocabulary of ancient peptides at the origin of folded proteins.* eLife, 2016. **4**: p. e09410.
